# Household environment and animal fecal contamination are critical modifiers of the gut microbiome and resistome in young children from rural Nicaragua

**DOI:** 10.1186/s40168-023-01636-5

**Published:** 2023-09-15

**Authors:** Molly Mills, Seungjun Lee, Barbara A. Piperata, Rebecca Garabed, Boseung Choi, Jiyoung Lee

**Affiliations:** 1 Division of Environmental Health Sciences, College of Public Health, The Ohio State University, Columbus, OH USA; 2 Environmental Sciences Graduate Program, The Ohio State University, Columbus, OH USA; 3 Department of Food Science and Nutrition, College of Fisheries Science, Pukyong National University, Busan, Republic of Korea; 4 Department of Anthropology, The Ohio State University, Columbus, OH USA; 5 Department of Veterinary Preventive Medicine, The Ohio State University, Columbus, OH USA; 6 Division of Big Data Science, Korea University, Sejong, Republic of Korea; 7 Department of Food Science & Technology, The Ohio State University, Columbus, OH USA

**Keywords:** Microbial source tracking, *E. coli* as antibiotic resistance host, Multi-drug resistance, Dirt floor, Animals, One Health, Breastfeeding duration

## Abstract

**Background:**

Early life plays a vital role in the development of the gut microbiome and subsequent health. While many factors that shape the gut microbiome have been described, including delivery mode, breastfeeding, and antibiotic use, the role of household environments is still unclear. Furthermore, the development of the gut antimicrobial resistome and its role in health and disease is not well characterized, particularly in settings with water insecurity and less sanitation infrastructure.

**Results:**

This study investigated the gut microbiome and resistome of infants and young children (ages 4 days-6 years) in rural Nicaragua using Oxford Nanopore Technology’s MinION long-read sequencing. Differences in gut microbiome diversity and antibiotic resistance gene (ARG) abundance were examined for associations with host factors (age, sex, height for age z-score, weight for height z-score, delivery mode, breastfeeding habits) and household environmental factors (animals inside the home, coliforms in drinking water, enteric pathogens in household floors, fecal microbial source tracking markers in household floors). We identified anticipated associations of higher gut microbiome diversity with participant age and vaginal delivery. However, novel to this study were the significant, positive associations between ruminant and dog fecal contamination of household floors and gut microbiome diversity. We also identified greater abundance of potential pathogens in the gut microbiomes of participants with higher fecal contamination on their household floors. Path analysis revealed that water quality and household floor contamination independently and significantly influenced gut microbiome diversity when controlling for age. These gut microbiome contained diverse resistome, dominated by multidrug, tetracycline, macrolide/lincosamide/streptogramin, and beta-lactam resistance. We found that the abundance of ARGs in the gut decreased with age. The bacterial hosts of ARGs were mainly from the family *Enterobacteriaceae*, particularly *Escherichia coli*.

**Conclusions:**

This study identified the role of household environmental contamination in the developing gut microbiome and resistome of young children and infants with a One Health perspective. We found significant relationships between host age, gut microbiome diversity, and the resistome. Understanding the impact of the household environment on the development of the resistome and microbiome in early life is essential to optimize the relationship between environmental exposure and human health.

Video Abstract

**Graphical Abstract:**

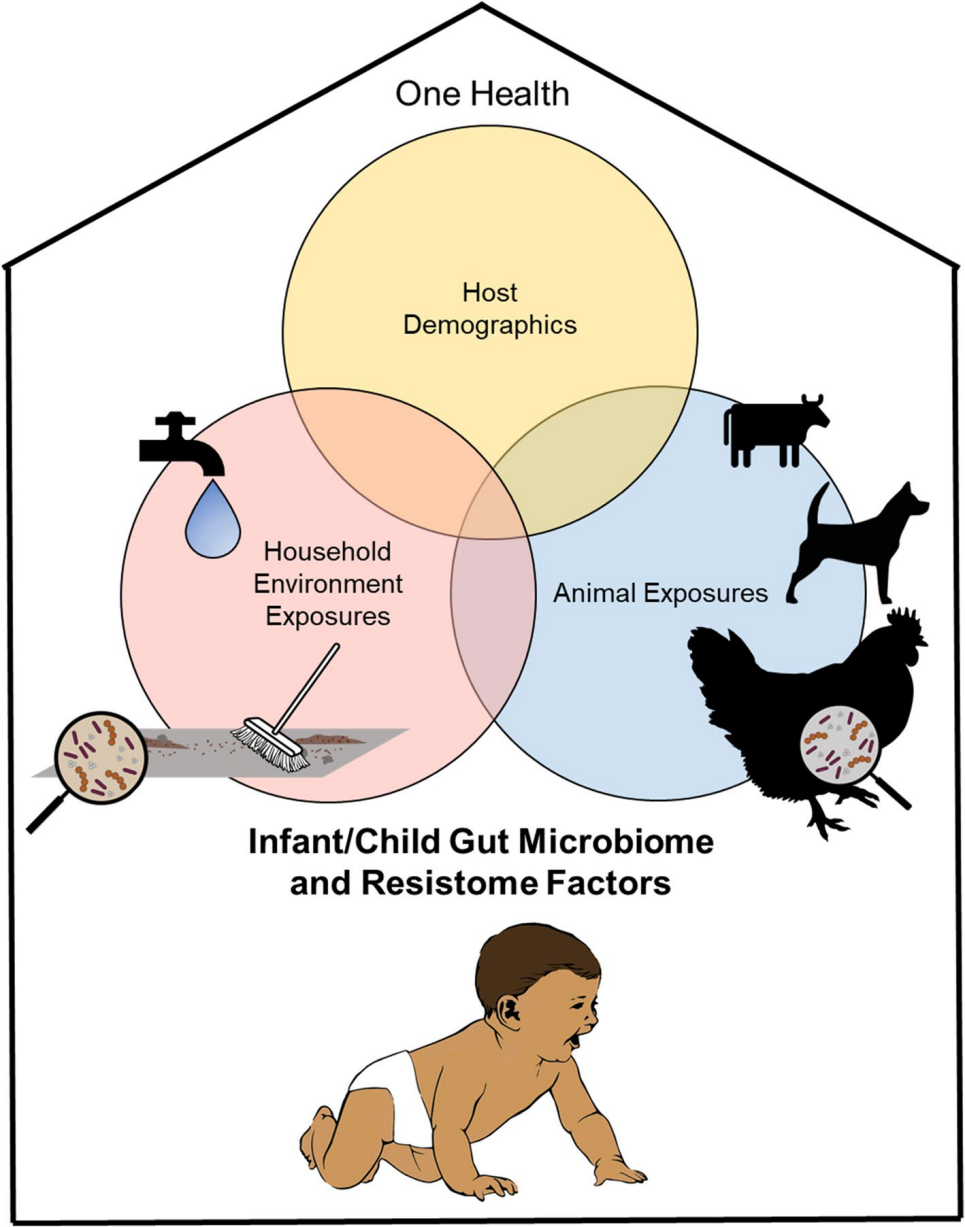

**Supplementary Information:**

The online version contains supplementary material available at 10.1186/s40168-023-01636-5.

## Background

Early life is a critical window in the development of the human gut microbiome and subsequent health [1]. The gut microbiome plays a critical role in maintaining and developing the immune system, the endocrine system, metabolism, and brain maturation and function [2–4]. It has been established that early life events shape the gut microbiome, including delivery mode (vaginal or cesarean), antibiotic exposure, feeding mode (breastfed or formula) and illness [1, 2, 5–7]. In general, the gut microbiome is affected by contact with surfaces, animals, and other people, ingestion of food/water, and inhalation that harbor various microorganisms [3]. Therefore, it is necessary to study the development of the gut microbiome using a One Health approach, which considers the relationships between human, animal, and environmental health [3]. Microbial exposures from both animals and the surrounding environment have the potential to modify human health via the gut microbiome.

The household environment, specifically, has been shown to play a major role in structuring the gut microbiome. For example, unrelated individuals who live in the same house have significant similarities in microbiome composition [8]. In recent history, there is a speculation that indoor environments have become cleaner, and sanitation has improved, which may be one of the leading contributors to the rise in allergic diseases [7]. Furthermore, there is evidence that this phenomenon, known as the hygiene hypothesis [9], is mediated by the gut microbiome [10, 11]. Fewer environmental exposures to microorganisms, particularly at a young age, may hinder gut microbiome development and maturation, preventing proper immune system functioning. Decreased gut microbiome diversity has been linked to immune-related diseases, including inflammatory bowel disease, allergies, asthma, and obesity [12]. However, not all microbial exposures are beneficial in early life. Animal exposures [13, 14] and rural farming environments [15] may have positive effects on gut microbiome diversity, but the pathogens found in animal feces can be considerable hazards to human health [16]. This is particularly a problem in low- and middle-income countries (LMICs), where there may be less separation of animal feces from the household environment [16]. Children from LMICs are exposed to more pathogens in their water, food, soil, and surfaces than children from higher-income countries, which puts this group particularly at risk [2].

The development of the human gut resistome, which is the collection of all the antibiotic resistance genes (ARGs) within a community [17], is not as well characterized as the microbiome [18]. However, the gut resistome has been related to nationality, sex, age, co-morbidities, gut microbiome composition, household environment, and daily exposures [19]. In children and infants specifically, it has been shown that taking oral antibiotics increases the richness [20] and abundance [21] of ARGs in the gut, but only over short time periods. This indicates that antibiotic exposure is not the only factor in the development of the resistome. The most likely sources of the resistome in early life are the mother, breast milk, and the environment [18, 21–23]. The gut resistome requires further study, particularly in infants and young children from LMICs, given the limited knowledge of the collection of ARGs in this sensitive group.

The main goal of this study was to characterize the gut microbiomes and resistomes of infants and young children (ages 4 days-6 years) from a rural community in Nicaragua with One Health perspective. Our prior publication analyzed the gut microbiomes of individuals from this population using 16S rRNA gene sequencing in the context of relative drinking water contamination [24]. However, in this study we use long read sequencing to characterize both the microbiome and resistome. This third-generation sequencing technique is beneficial in studies of the resistome because it enables identification of ARGs and their host microorganisms without assembly [25]. We also expand on our prior work in this study, as we incorporate a One Health approach. We analyze the gut microbiomes in the context of survey data (age, sex, delivery mode, breastfeeding practices), anthropometrics, drinking water quality, household environmental quality (contamination of indoor household floors), and chicken fecal samples (the most common domestic animal observed in the study sites). It is important to study antibiotic resistance (AR) from a One Health perspective in LMICs, given the challenges with sanitation and hygiene [26]. Our main hypotheses were that more contaminated household environments would be associated with greater gut microbiome diversity and resistome abundance in children and infants, according to the hygiene hypothesis. We also anticipated that more polluted environments would result in more opportunistic pathogens in the gut, due to the hazard associated with fecal contamination.

## Methods

### Sampling and household survey data collection

The sample collections and survey data collection were conducted in 2017 in Los Robles, Nicaragua. A thorough description of the community and households can be found in a prior publication, which analyzed gut diversity in relation to household water quality [24]. In summary, Los Robles is a farming community in the Department of Jinotega, one of the poorest regions of the country [27]. Most households raise livestock, such as chickens, pigs, and cows [24]. Houses in Los Robles are generally made of wood or cinderblock walls, metal sheeting for roofs, and packed dirt or cement for floors [27]. Most homes have access to electricity, but due to unstable access to drinking water, most households store drinking water in containers [24].

**Fig. 1:**
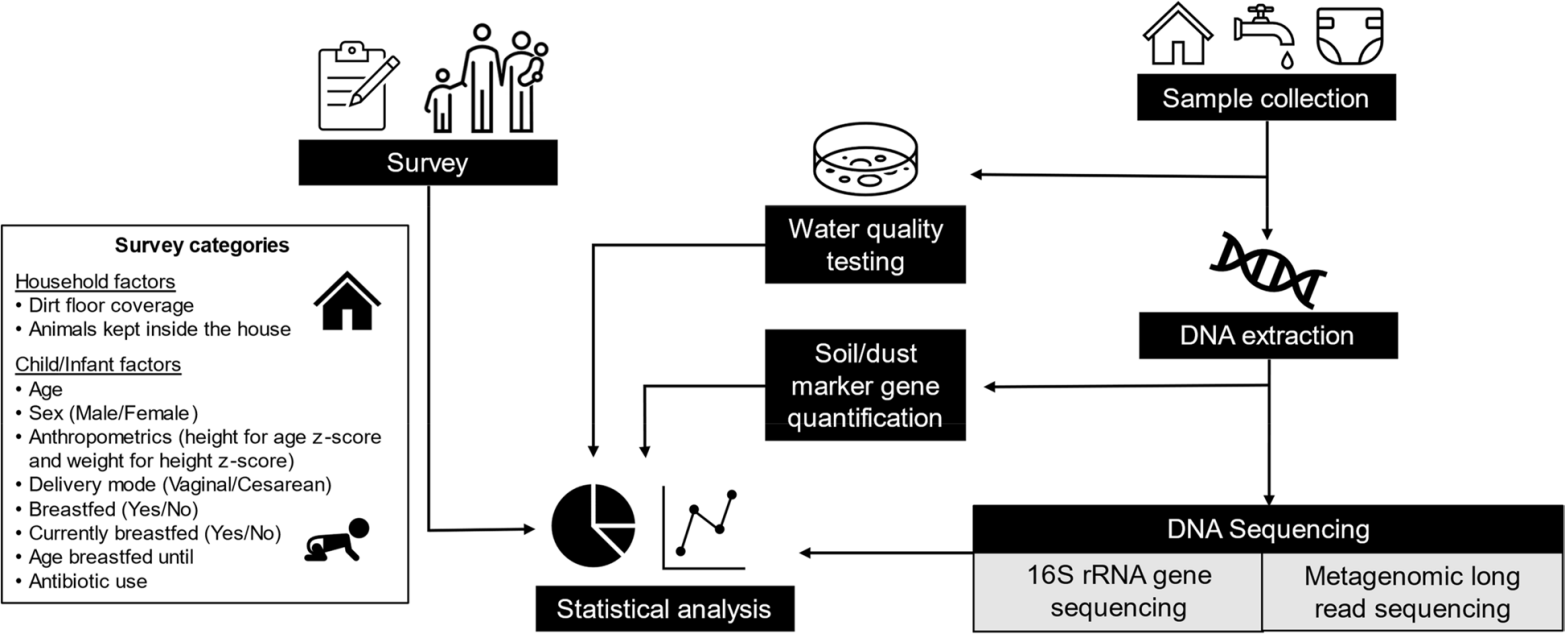
Conceptual diagram of study design. This study included a survey of the participants to gather information on the children/infants and observational data about each household. Household soil/dust and drinking water were collected from each house. Drinking water quality was tested on site. Fecal samples were collected from children, infants, and chickens. Household soil/dust was further analyzed via quantification of microbial source tracking (MST) and pathogenic marker genes. Chickens and a subset of household soil/dust samples were sent for 16S rRNA gene sequencing for microbial community analysis. Long-read metagenomic sequencing was completed for child/infant fecal samples. All inputs described were used in the final statistical analyses of the factors influencing the child/infant gut microbiomes and resistome

An overview of the study design and methods are shown in Fig. 1. Each household was visited once. During this visit, we administered a survey to the female head of household in which data were collected about each child in the household. The data collected included age, sex, delivery mode (vaginal or cesarean), breastfeeding (if they were ever breastfed (yes or no), breastfeeding duration, if they are currently being breasted (yes or no), and if the child had ever received antibiotics. We divided the participating children into age groups with those 0–1.99 years of age classified as infants and those between 2.0–6.0 years as children. To measure infant weight (g), we used a hanging spring scale. Children were weighed (kg) using a standard spring balance. Infant length was measured (cm) to the nearest 0.1 cm using a Seca 210 mobile measuring mat, and children’s standing height (cm) was measured to the nearest 0.1 cm using a Seca 213 portable stadiometer. Weights, heights, and lengths were measured twice, and the reported value was the average of the duplicate measures. In addition to the information on the children, we also used the survey to gather data on household floor type (all dirt, some dirt and some cement, all cement/other material) and if animals were allowed inside the home.

During the household visit, we collected two household environmental samples. A superficial soil/dust sample was collected from the most frequented indoor floor area by sweeping the surface and collecting the gathered dirt/dust. We also collected 400 mL of drinking water using sterile Whirl–Pak bags (Nasco, Fort Atkinson, Wisconsin), which we transported on ice to the laboratory. We filtered 200 mL drinking water through a sterile 0.22 μm EMD Millipore Isopore™ Polycarbonate Membrane Filter (Merck KGaA, Darmstadt, Germany). As chickens were abundant in and around the homes in this community, one chicken fecal sample was collected from each household with a child (2–6 years old), to represent the animal population that lives in proximity to the children. Chicken fecal samples were collected by either observing a chicken defecate and collecting the feces from the ground or placing the chicken in a plastic laundry basket and collecting the feces from the laundry basket. Chickens were not confined in the basket for more than 5 min. The collection of chicken fecal samples was reviewed and approved by the Ohio State University Institutional Animal Care and Use Committee (IACUC#: 2017A00000047). Fecal samples from children and infant diapers were collected to analyze the gut microbiome using the OMNIgene-GUT kit (DNA Genotek Inc., Ottawa, ON, Canada), which also stabilized the samples until being received by the laboratory for molecular analysis. To maintain the DNA quality of the chicken fecal, drinking water filter, and soil/dust samples, Allprotect Tissue Reagent (Qiagen, Valencia, California, USA) was added at the time of collection. In total, 31 fecal samples from children, 26 fecal samples from infants, 25 chicken fecal samples, and 2 household floor soil/dust samples from a total of 40 households were used in this study.

### Sample processing, DNA extraction, and molecular methods

We completed on-site coliform testing for household drinking water using 3 M Petrifilm *Escherichia coli* (*E. coli*)/coliform count plates (Petrifilm, St. Paul, Minnesota), per manufacturer’s instructions. We measured total coliforms in drinking water as the most probable number of colony forming units (CFU)/mL.

All DNA was extracted from human fecal, chicken fecal, drinking water, and household soil/dust samples via the QIAmp® Fast DNA Stool Mini Kit (Qiagen) with an additional bead-beating step (2 × 5 min at maximum speed- 150 × g). 200 mg of 0.1 and 0.5 mm diameter sterile Zirconia-silicate beads (Biospec Products Inc, Bartlesville, Oklahoma) were used to homogenize all samples with the Bead Mill 4 homogenizer (Fisher Scientific, Hampton, New Hampshire). DNA concentration and quality was measured for all samples using the NanoDrop™ 2000 Spectrophotometer (ThermoFisher Scientific, Waltham, Massachusetts (MA), USA).

We used Droplet Digital™ PCR (Bio-rad, Hercules, California) to quantify several target genes in household soil/dust samples. This included quantification of target genes for enteric pathogens (Shiga toxin-producing *Escherichia coli* (STEC), *Arcobacter sp.*, *Campylobacter sp.*, *Salmonella sp*.), and microbial source tracking (MST) markers (ruminant-specific gut bacteria (Rum2Bac), human-specific gut bacteria (HF183), and dog-specific gut bacteria (BacCan-UCD)) to characterize the contamination of the household floors. MST uses host-specific genetic markers to identify the sources of fecal contamination in a sample, so we selected human, dog, and ruminant (cattle) MST markers because of the animals observed in this community. We acknowledge that genetic MST markers for fecal identification and quantification may have altered specificity or abundance in different geographic locations [28]. However, the development of Nicaraguan-specific MST markers for the animals of interest in this analysis was outside the scope of this study. All Droplet Digital™ PCR methods, primer information, and results are described in the Supplementary information (Supplementary Methods; Table S1; Figure S1).

Chicken fecal, soil/dust, and human fecal DNA samples were purified and concentrated prior to sequencing. Fecal samples were cleaned and concentrated using a basic ethanol precipitation protocol, which can be found in the Supplementary information (Supplementary Methods). Soil/dust samples were cleaned and concentrated using the DNA Clean & Concentrator-100 kit (Zymo Research, Irvine, California, USA). Final DNA quality was measured using the NanoDrop™ 2000 Spectrophotometer (ThermoFisher Scientific), and DNA concentration was measured using a Qubit 3 Fluorometer (ThermoFisher Scientific) prior to sequencing.

The V4-V5 region of the 16S rRNA gene was sequenced from the chicken fecal and household soil/dust samples at the Molecular and Cellular Imaging Center (Wooster, OH) via the Illumina MiSeq platform*.* Only samples with DNA concentrations > 5 ng/µL and OD_260/280_ 1.8–2.0 were sent for 16S rRNA gene sequencing. This included 25/27 chicken fecal samples and 2/30 household soil/dust samples. Even following purification and concentration, household soil/dust samples had an average OD_260/280_ value of 1.66, limiting the number of samples with sufficient quality to sequence. We processed and analyzed the sequence data using the QIIME2 pipeline [29]. 16S rRNA gene sequencing and bioinformatics methodology is presented in the Supplementary information (Supplementary Methods). We sequenced the child and infant fecal samples using Oxford Nanopore Technology’s (ONT) long read MinION sequencing (ONT, Oxford, England). Thirty-one child and 26 infant fecal samples from 40 households were of suitable concentration and quality for metagenomic sequencing. We prepared and barcoded the sample libraries using the Rapid Barcoding Kit (SQK-RBK004, ONT). We sequenced the full dataset of 57 fecal samples on the Flongle flow cell (FLO-FLG001 R9.4.1). This library kit and flow cell allow for inputs of 200 ng DNA per sample. For analysis at a greater sequencing depth, a subset of 23 fecal samples were sequenced on MinION flow cells (FLO-MIN106d R9), which allows for inputs of 400 ng DNA per sample.

Long read sequence data were basecalled and demultiplexed using ONT’s basecalling program, Guppy (v 3.2.4), which is available at the ONT site (community.nanoporetech.com). We aligned genes and annotated taxonomy using ONT’s What’s In My Pot? (WIMP) tool, hosted on the cloud-based EPI2ME platform (2020.05.19). This workflow aligns and classifies reads against the National Center for Biotechnology Information (NCBI) taxonomy database. Taxonomy were paired with NCBI taxonomy IDs with TaxonKit [30]. We annotated ARGs, mobile genetic elements (MGEs), metal resistance genes (MRGs), and their taxonomic hosts using the tool NanoARG [31]. The reported results (ARG/MGE/MRG abundance) were normalized to each sample’s library size in average gene counts/gigabase pair (gbp), via NanoARG methods [31] which uses a similar approach as Ma et al. [32].

$${A}_{i}=\frac{{C}_{i}}{{C}_{g}(1)}$$

The normalized abundance (*A*_*i*_) is calculated by dividing *C*_*i*_, or the total count of the respective gene (ARG/MGE/MRG), *i*, by *C*_*g*_, or the size of the data in gbp [31]. We also include ARG annotations per sample using more strict cutoffs, via Resfinder [33] at 90% sequence identity and 60% minimum length for comparison to NanoARG [31], which uses more permissive parameters (E-value 1e − 5, identity 25%, coverage 40%, and –nk 15,000) [31] (Table S2). ResFinder results were not used in statistical testing, as there were low numbers of annotations in these samples, which limited resistome analysis (Table S2). In the Supplementary information, we also compare sequencing results between the Flongle and MinION flow cells (Table S3), as well as the microbiomes of children and infants using the different methods (Figure S2). All long read (Flongle and MinION flow cell) and 16S rRNA gene sequencing results and metadata can be found at the National Center for Biotechnology Information (NCBI) Sequence Read Archive (SRA) under the accession number PRJNA894152 (https://www.ncbi.nlm.nih.gov/sra/PRJNA894152).

### Statistical analyses

We carried out all statistical analyses in R (v 3.6.2). Anthropometric measures were calculated with the R package zscorer [34]. Length for age z-score and weight for length z-score were calculated for infants (< 2 years) based on length, weight, age, and sex. Height for age z-score and weight for height z-score were calculated for children (2–6 years) based on height, weight, age, and sex. We were interested in comparing microbiome diversity and resistome abundance with anthropometrics, as these are used to identify children’s nutritional status and development [35]. Given the diverse range of ages included in this study, it was important to include measures of development and growth, as well as a measure that could incorporate potential undernutrition, given the LMIC setting and known rates of undernutrition in children in this region [27].

For the microbiome/resistome analysis, data sequenced on Flongle flow cells were analyzed separately from data sequenced on MinION flow cells. We present the sequence data processing and normalization methods in the Supplementary information (Supplementary Methods). All α- and β-diversity indices were calculated using the R packages phyloseq [36] and vegan [37]. We measured α-diversity via Shannon index and Pielou’s evenness for microbiome analysis. All diversity indices were verified to not be significantly correlated with sequencing depth (*p* > 0.05). Furthermore, due to concerns over microbiome α-diversity indices being confounded with sequencing depth, all significant factors for microbiome α-diversity were verified to not be due to significant differences in sequencing depth (*p* > 0.05). All comparisons were not significant, except the comparison between 16S rRNA gene sequencing of chicken gut microbiomes and the long read sequencing of the infant and children gut microbiomes (*p* < 0.05). However, this difference was the opposite of that observed for the differences in microbiome α-diversity (chicken gut had significantly greater sequencing depth than human gut microbiomes). Therefore, we do not attribute the differences we observe in microbiome diversity to sequencing depth. Child and infant gut microbiome diversity measures are presented in the Supplementary information (Table S4). The resistomes of the child/infant fecal samples were analyzed based ARG abundance (NanoARG annotated normalized average ARG counts/gbp for all antibiotic classes). Traditional α-diversity indices such as Richness, Shannon index, and Pielou’s evenness were significantly associated with library size and sequencing depth due to the limited sequencing depth in these samples, so they were not used to describe the resistomes.

Resistome abundance and microbiome diversity indices were tested for differences by all survey data (age, sex, delivery mode, breastfeeding practices, animals kept inside the house, soil floor coverage, antibiotic use), calculated anthropometrics (height/length for age z-score, weight for height/length z-score), and environmental factors (coliform concentrations in drinking water, pathogens in household floors, MST marker gene concentrations in household floors). Microbiome α-diversity and ARG abundance were non-normal, as determined by Q-Q plots and Shapiro–Wilk tests. Therefore, Spearman correlations were used for all continuous variables, and Wilcoxon rank sum tests were used for all categorical metadata with two groups. Kruskal–Wallis tests were used for categorical variables with greater than two groups, followed by pairwise Wilcoxon rank sum tests with a Bonferroni adjusted *p*-value if the initial test was significant. We calculated β-diversity via Bray Curtis dissimilarity matrix. Permutational Multivariate Analysis of Variance (PERMANOVA) (adonis) tests from the vegan package [37] using distance matrices were conducted for each factor to determine significant differences in composition and by metadata variables in resistome and microbiome communities. Significance of factors were determined at *p* < 0.05. However, we also report factors with *p* < 0.1. Larger *p* values, even up to *p* < 0.2, can indicate a potential relationship in microbiome studies, and have previously been used as a threshold for significance [38, 39].

Differential abundance testing was conducted for factors with two groups using pairwise Linear discriminant analysis Effect Size (LEfSe) with the default settings, including normalization of the relative abundance values per sample to sum to 1 million (1 M), significance for overall and pairwise tests assessed at *p* < 0.05, linear discriminant analysis (LDA) effect size > 2.0, and a strict all-against-all comparison [40]. Subject age group (child vs. infant) was used as the sub-class in differential abundance testing by all categories. To determine differentially abundant taxa for continuous variables, we used Microbiome Multivariable Associations with Linear Models 2 (MaAsLin2) [41]. Taxa were identified in a model for all MST marker genes (ruminant, dog, and human fecal marker) and adjusted for subject age group (child v. infant). All factors were included as fixed effects and taxa were tested with a minimum abundance of 0.001 and a minimum prevalence of 0.2, based on prior literature that has used this method [42]. To determine the differentially abundant taxa with changes in MST marker gene concentrations, these factors were tested via MaAsLin2 as continuous variables using their concentrations in each sample (gene copies/g in household soil/dust) and LEfSe with the categories being presence and absence of the marker gene in household soil/dust.

Path analysis was conducted to determine the mediating effect of the environmental parameters between factors known to structure the developing gut microbiome (age, delivery mode) and gut microbiome diversity/resistome abundance. Environmental parameters (mediating effects) included a measure of household soil/dust contamination (soil score), which was the total number of MST markers and pathogens detected (0 or 1 for each marker gene that was detected) in household soil/dust via ddPCR, a measure of water contamination (water score), which was the total number of pathogenic gene markers detected in the household water via ddPCR, and a total measure of household environmental contamination (environmental score), which summed the soil/dust and water contamination scores. Eight separate models were fit for this data. The first four used Shannon index as the response variable, to test associations with gut microbiome diversity. The last four models used total ARG abundance as the response variable, to test associations with the gut resistome. Age and delivery mode (vaginal v. cesarean) were run in separate models as known, explanatory variables. Water and soil/dust contamination were tested as mediating effects in the same models; environmental score was tested separately, to determine the cumulative effect of the household environment.

## Results

### Gut microbiome diversity and composition are shaped by age and household contamination

**Fig. 2:**
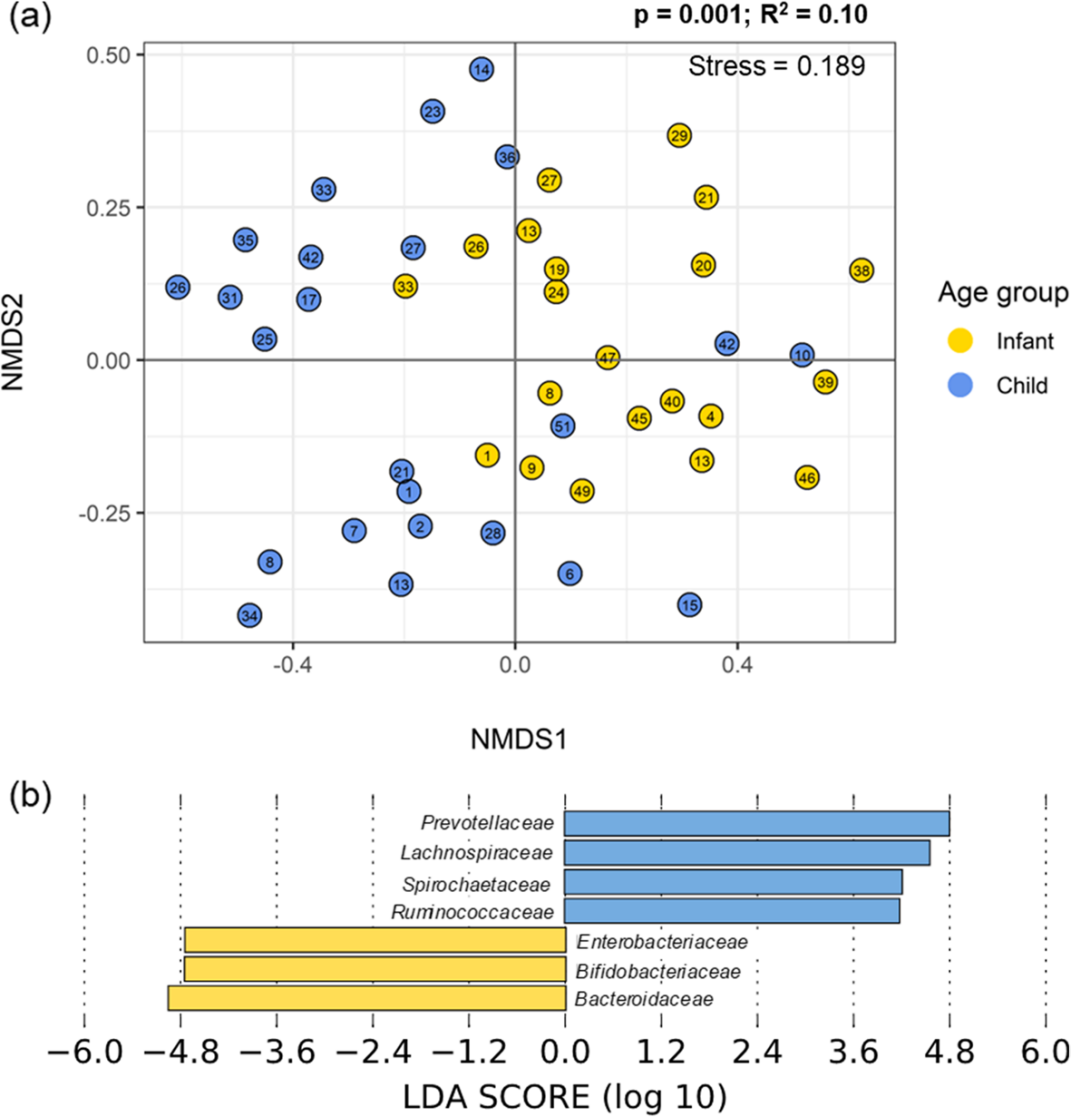
Differences in child and infant gut microbiomes. **a** Beta diversity of infants (yellow, ages 0–1.99) and children (blue, ages 2–6), plotted with Non-Metric Multi-dimensional Scaling (NMDS) ordination based on Bray Curtis dissimilarity. The number in each point indicates the household in which the sample was collected. The difference between children and infants was significant via PERMANOVA tests (*p* = 0.001); **b** Bacterial families with different abundances in children and infants were identified with linear discriminant analysis effect size (LEfSe). Families in blue were more abundant in children and families in yellow were more abundant in infants

**Fig. 3:**
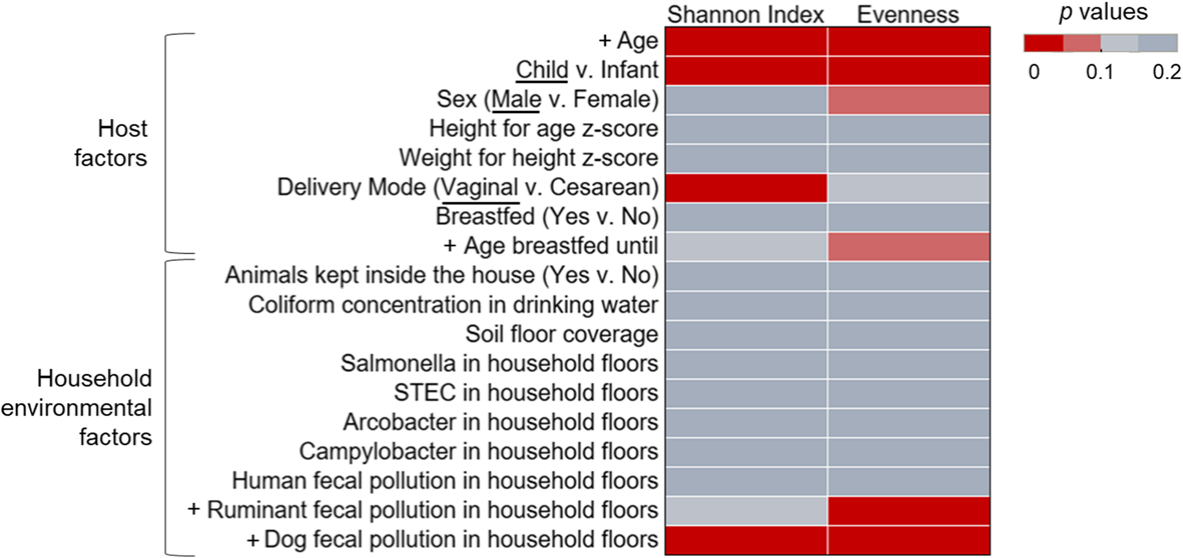
Significance of household environment and survey factors in gut microbiome diversity. The significance of the factors tested amongst the α diversity indices of Shannon index and Pielou’s evenness in children and infants. Significance was assessed via *p* value, with values differentiated by color. Significant values (*p* < 0.05) are shown with the darkest red. The direction of the trend is identified in the factor label (y-axis). For correlation tests of continuous variables, the direction of the significant association is indicated with a + or -. For categorical data, the group with significantly greater diversity is underlined

Gut microbiome α- and β-diversity in children and infants were the most different with age (Figs. 2–3). In particular, α-diversity increased with age (Shannon index (*p* < 0.001; *ρ* = 0.61), and evenness (*p* < 0.001; *ρ* = 0.62)) (Fig. 3). As a result, there were also significant differences in gut microbiome α-diversity between children and infants (Shannon index (*p* < 0.001), evenness (*p* < 0.01)) (Fig. 3; Figure S3a). There were significant differences in β-diversity of the gut microbiome communities with age (*p* = 0.001) and between infants and children (*p* = 0.001) (Fig. 2a), highlighting the difference in microbial community structure and composition with subject age. Comparing infants and children, we found the abundances of seven families to be significantly different (Fig. 2b). *Enterobacteriaceae*, *Bifidobacteriaceae*, and *Bacteroidaceae* were more abundant in infant gut microbiomes. *Prevotellaceae*, *Lachnospiraceae*, *Spirochaetaceae*, and *Ruminococcaceae* were more abundant in child gut microbiomes.

The other host factor that was significantly associated with gut microbiome diversity was delivery mode (vaginal v. cesarean)(Fig. 3). The Shannon indices of the gut microbiomes of individuals delivered vaginally were greater than those delivered via cesarean section (*p* < 0.05) (Fig. 3; Figure S3b). Furthermore, male infants and children had near significantly greater gut microbiome evenness than females (*p* = 0.053). Finally, while not significantly different, the age infants and children were breastfed until was positively associated with gut microbiome evenness (*p* = 0.098; *ρ* = 0.31) (Figure S4a). Data on antibiotic use was only available for a subset of *n* = 11, which limited the power of any findings. However, these results are presented in the Supplementary information (Figure S5). There were no significant differences with gut microbiome diversity and anthropometrics (height for age z-score, weight for height z-score)(Fig. 3).

Among the household environmental factors, the concentration of ruminant fecal marker in household soil/dust was positively correlated with infant and child gut microbiome evenness (*p* < 0.05; *ρ* = 0.36) (Fig. 3; Figure S4b). The concentration of a dog fecal marker in household soil/dust was positively correlated with gut microbiome Shannon index (*p* < 0.05; *ρ* = 0.41) and evenness (*p* < 0.05; *ρ* = 0.40) (Fig. 3; Figure S4b). Ruminant and dog fecal marker concentrations were not associated with age, as that would indicate confounding (*p* > 0.05). Differentially abundant taxa were identified in the infant and child gut microbiomes with changes in the ruminant and dog fecal marker genes in household soil/dust (Table 1). The relative abundance of *Clostridium perfringens*, *Streptococcus infantarius*, *Campylobacter jejuni*, unclassified *Anaerostipes*, *Anaerostipes hadrus*, *B. salanitronis*, *B. cellulosilyticus*, *Bacteroides helcogenes*, *Clostridium bolteae*, unclassified *Campylobacterales*, and unspecified bacteria in the gut increased with ruminant fecal marker gene concentration in household soil/dust. *Barnesiella viscericola* was more abundant in infant and child gut microbiomes with increased dog fecal marker gene concentrations in the household soil/dust. *Parabacteroides distasonis*, unclassified *Parabacteroides*, and *Lachnoclostridium phocaeense* were less abundant in the gut microbiomes of those living in homes with higher concentrations of dog fecal marker genes in household soil/dust samples.

**Table 1:** Several differentially abundant taxa were associated with the ruminant-associated and dog-associated fecal genetic marker abundance in household soil/dust. The taxa that were more abundant with the microbial source tracking (MST) marker gene (top row) are differentiated from those that were less abundant with the MST gene (bottom row). An asterisk (*) indicates significance identified via Microbiome Multivariable Associations with Linear Models (MaAsLin2) testing, which was used for continuous variables. Those taxa that are not marked were identified via linear discriminant analysis effect size (LEfSe), using 2 groups, presence or absence of the marker gene

	Ruminant-associate fecal marker	Dog-associated fecal marker
Taxa abundance increased with MST gene	Clostridium perfringens*, Streptococcus infantarius*, Campylobacter jejuni*, *Anaerostipes*, *Anaerostipes hadrus*, *Bacteroides salanitronis*, *Bacteroides cellulosilyticus*, *Bacteroides helcogenes*, *Clostridium bolteae*, Campylobacterales, Unspecified Bacteria	Barnesiella viscericola*
Taxa abundance decreased with MST gene	NA	*Parabacteroides distasonis*, *Parabacteroides*, *Lachnoclostridium phocaeense*

This analysis of host and household environmental factors was repeated for α-diversity indices (Shannon index, and evenness) in infant and child gut samples separately, which is presented in the Supplementary information (Figure S6). Overall, there were minimal associations with the α-diversity of the infant gut microbiome (Figure S6a). There was a significant, positive association between children’s gut microbiome evenness and age (*p* < 0.05; *ρ* = 0.60). Viral and fungal community analyses were limited due to the shallow sequencing depth, but their results for all participants are presented in the Supplementary information (Supplementary Results; Figures S7-S9).

### Environmental, animal gut, and human gut microbial communities differ in diversity

**Fig. 4:**
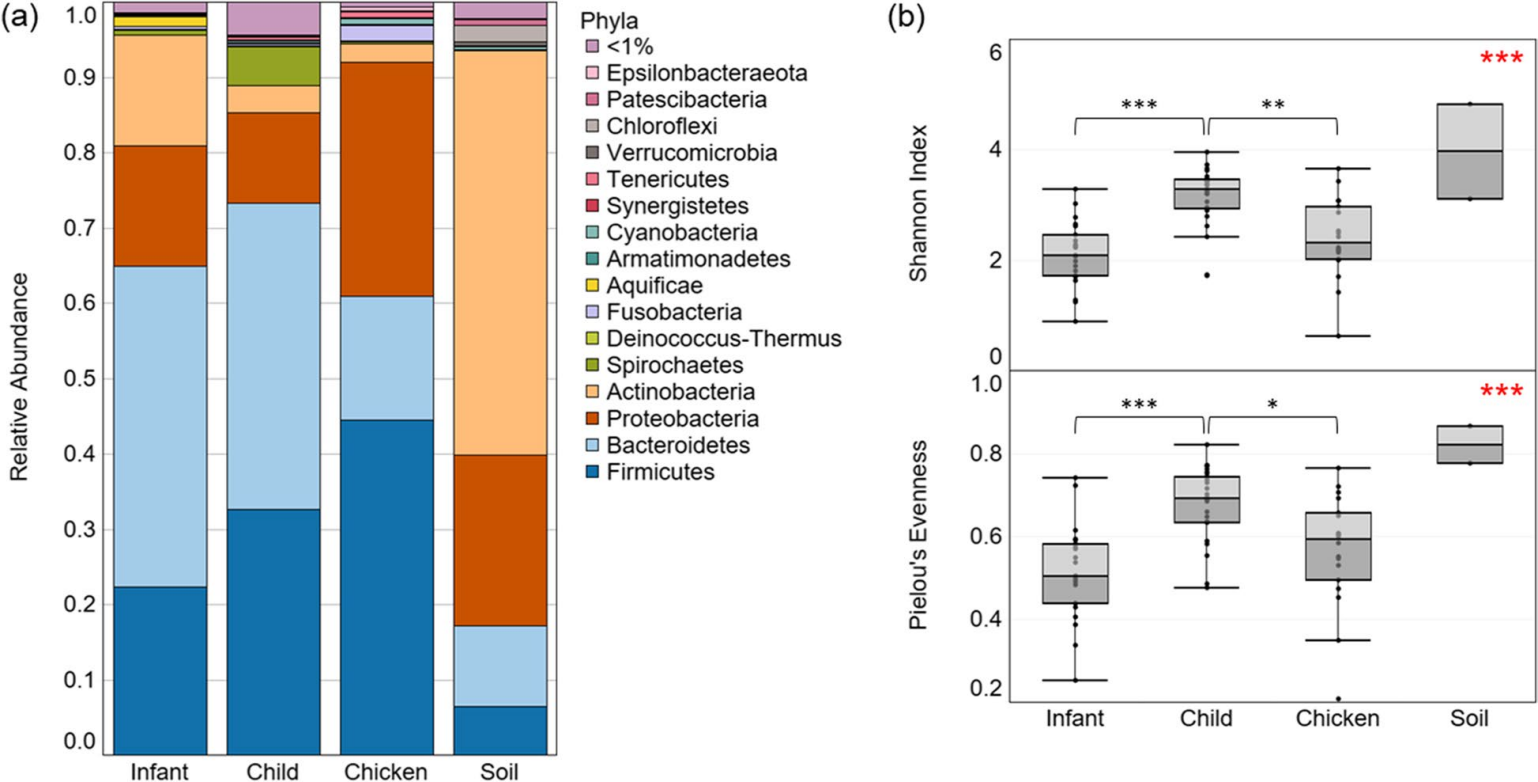
Comparison of infant, children, and chicken gut microbiomes and household soil/dust microbiomes. **a** The microbial community composition of infant, child, and chicken guts, as well as household soil/dust samples are shown as relative abundance at the phyla level of taxonomy; **b** α diversity is shown in all sample types, as Shannon index and Pielou’s evenness. Overall significance, assessed with Kruskal–Wallis tests, is indicated in red in the top right corner of each figure (comparison between infant, children, and chicken gut microbiomes). Subsequent pairwise Wilcoxon rank sum tests with a Bonferroni adjusted *p* value were completed between groups, and significance is indicated with black brackets and asterisks. *p < 0.05, **p < 0.01, ***p < 0.001

The most abundant phyla differed between the environmental, animal gut, and human gut microbiomes (Fig. 4a). The gut microbiomes of both infants and children were dominated by *Bacteroidetes* and *Firmicutes*, composing over 60% of the total microbial community. The next most abundant phylum was *Proteobacteria*. The most abundant phyla in the chicken gut microbiomes were *Firmicutes* and *Proteobacteria*, which combined composed over 75% of the community. The next most abundant phylum in the chicken gut was *Bacteroidetes*. *Actinobacteria* (53%) dominated in the soil/dust samples, with the next most abundant phyla being *Proteobacteria* and *Bacteroidetes*. Due to the limited sample size of soil samples (*n* = 2), differences in the abundances of taxa were not tested between environmental, human gut, and animal gut microbiomes.

At a family level, infant gut microbiomes were dominated by *Bacteroidaceae* (34.2%), whereas the child gut microbiome had more even distribution of the most abundant families, which included *Bacteroidaceae* (18.5%), *Lachnospiraceae* (16.0%), and *Prevotellaceae* (13.5%) (Table 2). Chicken gut microbiome samples were dominated by *Enterobacteriaceae* (21.8%), but *Lactobacillaceae* (12.4%) were also in high abundance. The top two families in household soil/dust samples were *Intrasporangiaceae* (15.8%) and *Micrococcaceae* (14.2%).

**Table 2:** The average relative abundance of the 10 most abundant families within microbial communities by sample type

	Baby	%	Child	%	Soil	%	Chicken	%
1	*Bacteroidaceae*	34.2	*Bacteroidaceae*	18.5	*Intrasporangiaceae*	15.8	*Enterobacteriaceae*	21.8
2	*Bifidobacteriaceae*	12.9	*Lachnospiraceae*	16.0	*Micrococcaceae*	14.2	*Lactobacillaceae*	12.4
3	*Enterobacteriaceae*	13.4	*Prevotellaceae*	13.5	*Dermabacteraceae*	4.6	*Lachnospiraceae*	6.6
4	*Lachnospiraceae*	8.5	*Ruminococcaceae*	5.9	*Halomonadaceae*	4.3	*Clostridiaceae 1*	6.2
5	*Ruminococcaceae*	4.0	*Enterobacteriaceae*	3.7	*Corynebacteriaceae*	3.1	*Bacteroidaceae*	4.7
6	*Prevotellaceae*	3.8	*Spirochaetaceae*	3.4	*Flavobacteriaceae*	2.6	*Prevotellaceae*	3.9
7	*Streptococcaceae*	3.5	*Bifidobacteriaceae*	2.7	*Nocardioidaceae*	2.3	*Ruminococcaceae*	3.5
8	*Veillonellaceae*	2.3	*Clostridiaceae*	1.8	*Xanthomonadaceae*	2.1	*Muribaculaceae*	2.8
9	*Tannerellaceae*	1.4	*Odoribacteraceae*	1.7	*Cyclobacteriaceae*	2.0	*Peptostreptococcaceae*	2.6
10	*Brachyspiraceae*	1.2	*Rikenellaceae*	1.7	*Idiomarinaceae*	1.9	*Moraxellaceae*	2.6

We tested differences between the α-diversity of the chicken, child, and infant gut microbial communities. Soil was not included due to the limited sample size (*n* = 2). There were significant differences in microbial community Shannon index (*p* < 0.001) and evenness (*p* < 0.001) (Fig. 4b). All α-diversity indices were greater in children than in infants or chickens (*p* < 0.05).

### The child and infant gut resistome contain a variety of ARGs

The infant and child gut microbiome hosted a diverse collection of ARGs from different antibiotic classes. The most abundant ARGs in both infants and children were for multidrug resistance, meaning these genes carry resistance mechanisms that are effective against multiple antibiotic classes (Figure S10). In infants, the next most abundant ARGs by antibiotic classes were tetracyclines and macrolide/lincosamide/streptogramins (MLS). Among children, the next most abundant ARGs by antibiotic class were glycopeptides and tetracyclines. The most abundant ARGs in infant’s and children’s guts are presented in the Supplementary information (Table S5).

**Fig. 5:**
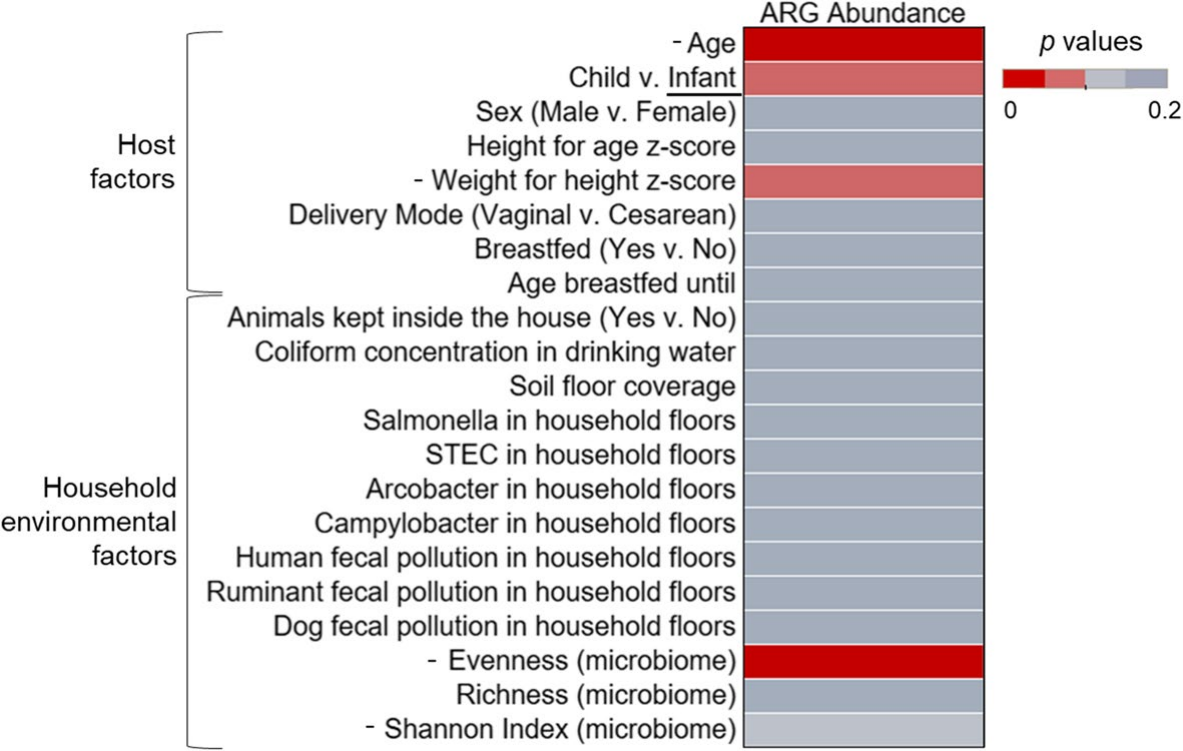
Significance of household environment and survey factors in gut resistome abundance. The comparison of the significance of different factors in describing total antibiotic resistance gene (ARG) abundance (normalized ARG counts/gigabase pair) in children and infants. Significance was assessed with *p* values, which are indicated by color. Significant values (*p* < 0.05) are shown with the darkest red. For significant factors, the direction of the trend is identified in the factor label (y-axis). For correlation tests of continuous variables, the direction of the significant association is indicated with a + or -. For categorical data, the group with significantly greater diversity is underlined. All cells shaded with the darkest grey had a *p* value > 0.15 (not significant, not reported)

The abundance of ARGs in the gut (the normalized total ARG counts/gbp per sample) decreased with age (*p* < 0.05; *ρ* = -0.37) (Fig. 5; Figure S11b). The difference in ARG abundance between children and infants was not significant, but it was close to our threshold of *p* < 0.05 (*p* = 0.061) (Fig. 5; Figure S11a). β-diversity analysis of gut resistome composition revealed no differences between infants and children (*p* > 0.05) (Figure S11c).

The same host and environmental factors in the gut microbiome analysis were used to test differences in ARG abundance. Weight for height z-score was not significantly associated with ARG abundance, but the *p* value was less than 0.1 (*p* = 0.097; *ρ* = -0.26) (Fig. 5; Figure S12). The differences in ARG abundance were also correlated with microbiome α-diversity indices. Microbiome evenness was negatively correlated with ARG abundance (*p* < 0.01; *ρ* = -0.42) (Fig. 5; Figure S12). Children and infant gut resistomes were analyzed separately as well, which is presented in the Supplementary information (Figure S13). Of note, children alone had the same trends in their resistomes as children and infants combined; gut microbiome evenness was negatively associated with ARG abundance (*p* < 0.05; *ρ* = -0.45). There were no further significant differences in ARG abundance in the guts of infants and children with these factors, when tested together or separately (*p* > 0.05).

**Fig. 6:**
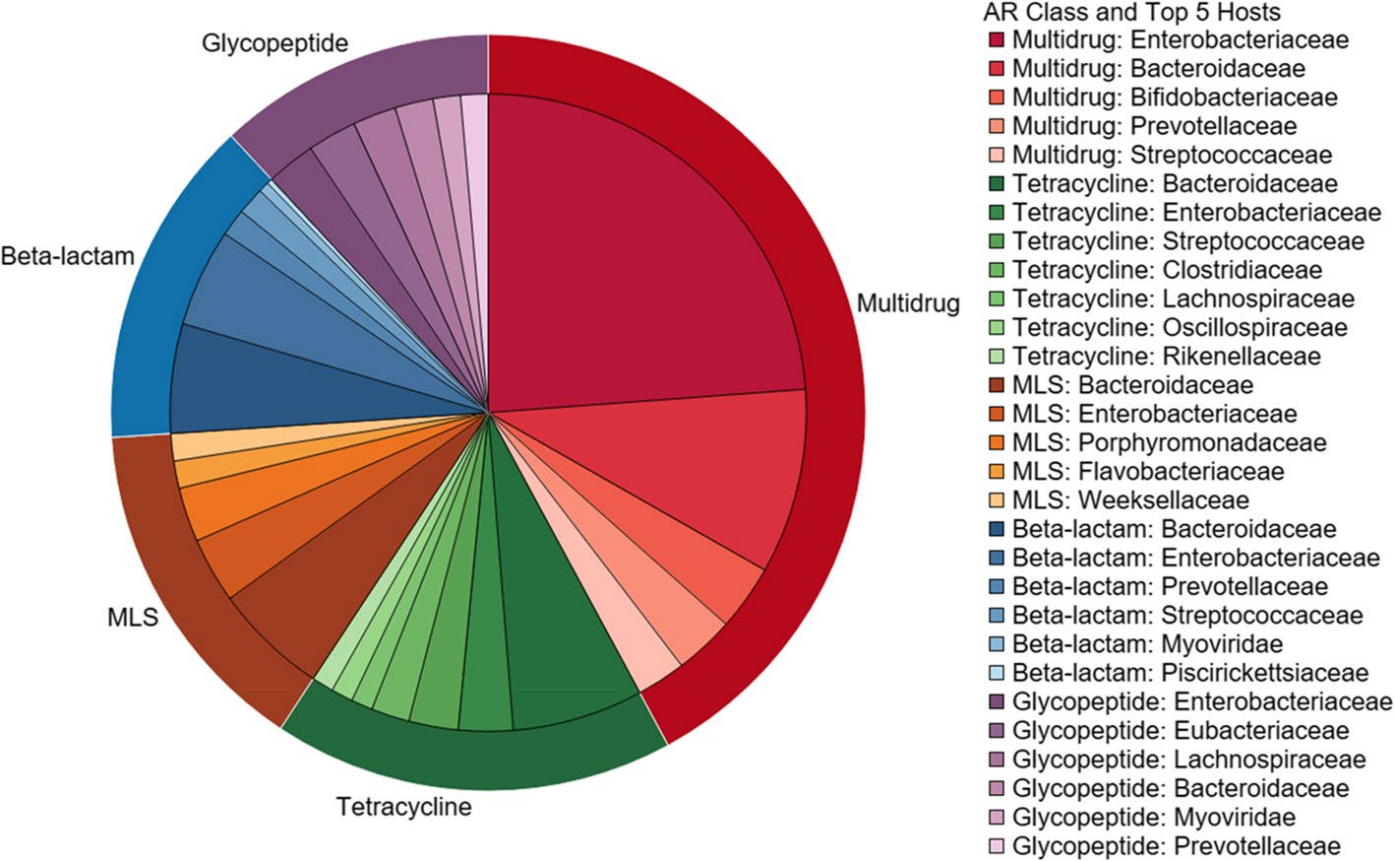
Antibiotic resistance gene class and respective microbial hosts in infant and children guts. The top five most abundant classes of antibiotic resistant genes in all children and infant guts are shown on the outer ring. Within each antibiotic class, the top five most abundant microbial hosts are identified at a family level

The most abundant identified bacterial host of ARGs in this study was *E. coli* (14.0%), followed by *Bacteroides salanitronis* (2.65%), *Bacteroides fragilis* (2.55%), and *Eubacterium rectale* (2.25%) (Figure S14). Among the ten most common bacterial host species, one was identified as a putative pathogen, *Klebsiella pneumoniae* (*K. pneumoniae*). Two viruses were among the ten most abundant ARG host species in the guts of children and infants, which included *Enterobacteria phage SfMu* and *Escherichia virus mu*. At a family level, some differences can be observed between ARG classes’ most frequent hosts (Fig. 6). The most abundant hosts for multidrug resistance were from the family *Enterobacteriaceae*. However, the most common host family for tetracycline, MLS, and beta-lactam ARGs was *Bacteroidaceae*. The most abundant host families for glycopeptide ARGs were *Enterobacteriaceae* and *Eubacteriaceae*. The viral family *Myoviridae* was in the five most abundant hosts for the beta-lactam and glycopeptide ARG classes.

Metal resistance genes (MRGs) and mobile genetic elements (MGEs) were also annotated in the sequence data from the child and infant guts, and the abundance of these genes can be found in the Supplementary information (Figures S15-S16). While not formally analyzed here, heavy metals have been shown to have a role in antimicrobial resistance, survival of bacteria in the gut, and child health, so these genes may merit further exploration.

### Environmental contamination independently influences the microbiome and resistome when controlling for age

In the gut microbiome diversity models, drinking water and soil/dust contamination had no significant mediating effects on the relationship between age and gut microbiome diversity (Shannon index), when tested together (environmental score) and separately (water score + soil score) (Table S7-S8). However, when controlling for age, household soil/dust and drinking water contamination significantly influenced gut microbiome diversity (*p* < 0.05), indicating the independent effect of the household soil/dust contamination and drinking water quality on the gut microbiome. We found that total environmental contamination (water + soil/dust) had a mediating effect on the relationship between birth type (vaginal v. cesarean) and Shannon index (Table S9-10). However, this environmental parameter had a complete mediating effect on the relationship between birth type and gut microbiome diversity, as the direct effect of birth type was not significant when adding the environmental parameter in the model. Finally, in the gut resistome models, no environmental parameters were found to mediate the relationships between age or birth type and total ARG abundance (Tables S11-12). However, when controlling for age, household soil/dust contamination slightly influenced ARG abundance in the gut (*p* = 0.087) (Table S11). Otherwise, there were no significant relationships between drinking water or household floor contamination and ARG abundance.

## Discussion

### Early life gut microbiomes are structured by age and delivery mode

Overwhelmingly, the strongest factor in describing differences in gut microbiome diversity and composition was subject age, as expected (Fig. 3). This study confirms that infants have lower gut microbiome diversity than older children, which has been well documented [1, 2]. Generally, around age 3, after weaning and the introduction of solid foods, the gut microbiome stabilizes and resembles an adult [7], but this depends on when solid foods are given. Our results also demonstrated that the differentially abundant bacterial families between infant and child gut microbiomes are likely driven by developmental and dietary changes. *Bifidobacteriaceae* and *Bacteroidaceae* were more abundant in infants, and genera from these families, such as *Bifidobacterium*, *Bacteroides*, and *Clostridium*, are known to be first colonizers of the human gut [2]. Bacteria within the family *Enterobacteriaceae* are also known to be abundant in the infant gut and important in early gut colonization, particularly *E. coli* [43, 44]. *Bifidobacterium*, represented here by their family *Bifidobacteriaceae*, are crucial in the infant gut because they break down human milk oligosaccharides (HMOs) to short chain fatty acids (SCFAs), which play an important role in gut health [45, 46]. More diverse microorganisms are necessary to break down more complex macromolecules because of the dietary changes that come with weaning, which may explain the increased abundances of *Prevotellaceae*, *Lachnosipraceae*, *Spirochaetaceae*, and *Ruminococcaceae* in the children in this study [7].

Gut microbiome diversity (Shannon index) of infants and children was significantly different with delivery mode, which is surprising, since studies have found that the differences in gut microbial communities due to delivery mode decrease with time, even over the first year of life [5, 47]. However, our path analysis revealed that the association between delivery mode and gut microbiome diversity (Shannon index) was fully mediated by the measure of total household environmental contamination (water + soil/dust) (Table S10), indicating that delivery mode does not have a direct effect on gut microbiome diversity in this study population, but this is only a reflection of the effect of environmental contamination on the gut. We suggest that a further study will be needed to more accurately determine whether the household environment and choice in delivery method might be linked culturally in this region and its potential to be confounding.

### Household fecal contamination may increase microbiome diversity but pose health risks

Even though humans are continuously in contact with the environment, few studies have focused on the relationship between environmental factors, especially contamination and its source, and the gut microbiome. Our results show that household environmental contamination has less of an influence on the gut microbiome than age. However, higher concentrations of fecal genetic markers for ruminants (likely cattle, based on the research team’s on-site observations) and dogs were linked to increased gut microbiome diversity.

Prior studies measuring animal exposures have asserted the hygiene hypothesis [13, 14, 48]. Specifically, contact with animals has been shown to increase gut microbiome diversity [13], including participating in livestock feeding chores [14] and pet ownership [48]. In general, it has been shown that the prevalence of allergies and asthma among children living on farms is lower compared to those living in urban contexts [49, 50], which provides evidence for the association between increased gut microbiome diversity and immune system development. In our study, animal exposures were measured indirectly as presence of animal feces in the household environment. These findings support our original hypothesis that greater gut diversity would be positively associated with household contamination.

Gut microbiome diversity was not significantly different with drinking water quality in our correlation analyses (Fig. 3), which differs from our prior study of this population [24]. Using 16S rRNA gene analysis, we found lower gut microbiome diversity among infants and children with higher concentrations of total coliforms in their drinking water [24]. In the current study, we found that both drinking water contamination (total coliforms) and household floor contamination (pathogens and MST markers) significantly influence Shannon index of the gut microbiome when controlling for age in the path analysis (Tables S7). This demonstrates the value of including multiple aspects of the environment when studying the microbiome, compared to the prior study which only included drinking water quality.

There were beneficial and potentially harmful microbes in the gut microbiomes that changed in abundances with the animal fecal contamination of household floors. The taxa that were more abundant in the gut with increased ruminant feces were likely due to contact with cattle feces being carried in from outdoors. These taxa were mostly from the families *Clostridiaceae*, *Bacteroidaceae*, and *Lachnospiraceae*, which have been ubiquitously identified in metagenomic analysis of cattle feces [51]. *Campylobacter* are also found in cattle feces [51, 52]. *Clostridium perfringens*, *S. infantarius*, and *Campylobacter jejuni* have been implicated as potential pathogens [53–55]. This highlights the obvious risk of enteric infection from household environments contaminated with ruminant feces. *Anaerostipes* spp. and *A. hadrus* are a common commensal of the human gut that produce butyrate, which provides anti-inflammatory properties, maintains gut homeostasis, and can help to maintain the gut barrier [56, 57]. *Anaerostipes* spp. are also found in both ruminants and chickens. It is interesting that a beneficial microbe is more abundant in the guts of infants and children with greater concentrations of animal fecal contamination, because this may functionally explain the increased gut microbiome evenness with ruminant fecal loading. Finally, the last three taxa that were more abundant with ruminant fecal contamination of household soil/dust were three species of *Bacteroides* that are commensals, but generally host-specific. *B. salanitronis* is adapted to chickens [58], *B. cellulosilyticus* is likely adapted to human guts [59], and *B. helcogenes* is thought to be adapted to pigs [60]. The identification of three host-specific strains of *Bacteroides* in the human gut, two of which are from animals that were present in and around houses in this community, may indicate further sharing of microbes between humans and domesticated animals in this setting.

The taxa that were identified as changing in abundance with the dog fecal marker in the soil/dust had more contradictory implications. While *B. viscericola* was first identified from a chicken gut, and their role is not well-described in the human gut [61]. However, the genus *Barnesiella* is probiotic and inversely related to vancomycin-resistant *Enterococcus* (VRE) in the mouse gut, and thus potentially beneficial against AR [62]. *B. viscericola* may help to explain the increased diversity in these communities, but more research is needed on this species in the gut to determine its relationship with fecal contamination. Both unclassified *Parabacteroides* and *P. distanosis* were decreased in the gut with increased dog fecal loading of household soil/dust. Common in the human gut, *Parabacteroides* spp. are anti-inflammatory commensals [63]. *P. distanosis* has shown benefits in controlled mouse studies resulting in metabolic benefits, such as decreased weight gain, hypoerglycemia, and elevated bile acids [64]. The reduction of these beneficial bacteria in the gut with dog fecal pollution of household soil/dust may have health implications.

Certain taxa may be increased in child and infant guts due to direct microbial transfer of zoonotic bacteria from the household soil/dust themselves, or more indirect mechanisms [3]. Housing animals in human living quarters is associated with increased risk of diarrhea, particularly from chickens [55]. Several households in this study were observed to keep chickens indoors, including in and around sleeping areas. The health risks from poor animal and sanitation containment can be significant, particularly for children and infants [55]. Benefits of appropriate animal exposure could still be maintained with proper sanitation in households, to maximize beneficial microbial exposures while preventing illness. The hygiene hypothesis presents unique and complex challenges that are amplified in rural household contexts.

### A One Health perspective lends insight into human gut microbial community diversity

Overall, the human gut microbiomes were compared to household soil/dust and chicken gut microbial communities. The general similarity between chicken and human gut microbiome composition that can be observed has been reported in prior study, which identified that the human and chicken gut can share up to 10% of the same genes [65]. In an analysis of over 600 chicken gut microbiome samples from 10 studies and 12 countries, the most frequently identified phyla were *Firmicutes*, *Bacteroidetes*, *Actinobacteria*, and *Proteobacteria* [66], which are represented in this analysis. A limitation of this analysis was the ability to only sequence soil samples from two households, due to the limited DNA concentrations. These two soil microbial communities had different compositions, so the composite average represented in this analysis does not represent all the households in this study (Table S6).

Similar differences in diversity of environmental, animal gut, and human gut microbiomes have been described. A prior study in rural Kenya also found that children (≤ 5 years) had greater gut microbiome diversity than chickens, while household environmental swab samples from the floor of the living space and cooking area had the greatest diversity when compared to animal and human guts [14]. In this study, children had greater gut microbiome diversity than chickens, but infants did not (Fig. 4b), which is an interesting distinction. More age-inclusive studies have found that adult and child gut microbiomes are less diverse than microbial communities from outdoor environmental matrices and domesticated animals [67].

### Nicaraguan infants and children carry a diverse resistome

Infants and young children in Los Robles carry a diverse collection of ARGs that confer resistance to many different antibiotic classes. The resistome of Nicaraguan infants/children have not previously been characterized to this extent. Two prior studies of Nicaraguan children < 5 years only determined AR phenotypically via culturable *E. coli* isolates [68, 69]. Furthermore, resistance testing was targeted for the set of antibiotics that isolates were tested against, which is limited compared to the range of ARGs and classes in the database used in this study. These prior studies found that most resistant isolates were for tetracyclines, ampicillin (a beta-lactam), and trimethroprim-sulfamethoxazole (a sulfonamide) [68, 69]. Comparatively, tetracycline and beta-lactam ARGs were abundant in the guts of infants and children in this study. Sulfonamide resistance, while present, was minimal in comparison to other classes (Figure S10). While studies in other countries may have used more similar methods to this study, the resistome has been established as highly dependent on the country of origin [70], so comparison may not be appropriate.

An important factor related to resistome development is gut microbiome maturity. More mature gut microbiomes have diminished ARG abundance, as demonstrated in infants (1 year) [21]. Gut microbiome maturity and diversity related to many of the factors described in this resistome analysis. For example, ARG abundance significantly decreased with age. It has been hypothesized that during early life, ARG abundance decreases as a subjects get older until AR abundance stabilizes [21]. This trend of ARG abundance decreasing with age has been seen in the first year of life in a prior longitudinal study in Luxembourg [71]. Other studies comparing the infant gut resistome to that of mothers show that infants carry significantly more ARGs, even without antibiotic use [23]. This phenomenon of greater ARGs in infant guts is hypothesized to be due to the early colonizers and members of the infant gut being common ARG hosts, such as *E. coli*, as well as their overall low gut microbiome diversity [21, 23]. This relationship between ARG abundance and gut microbiome maturity was also verified in the significant association between increased gut microbiome evenness and decreased ARG abundance (Fig. 5). While statistically not significant, we also observed a negative association (*p* = 0.097; *ρ* = -0.26) between weight for height z-score and ARG abundance, which is a metric that is used to identify children’s nutritional status [72]. Weight for height z-score has been positively associated with gut microbiome diversity [73], however we did not observe any significant differences with gut microbiome diversity in this analysis (Fig. 3; Figure S6). Our findings highlight the potential for relationships between nutritional status and the gut resistome, however further study is necessary to elucidate this connection.

In the MinION flow cell analysis, which had greater ARG abundance and a greater sequencing depth than the Flongle flow cell analysis, there were no significant trends between microbiome α-diversity indices and total ARG abundance (Table S13). However, the abundance of ARGs for multidrug resistance, the top ARG class, has a significant, negative correlation with Shannon index and evenness of the gut microbiome (*p* < 0.05) (Table S13). We also found a significant, negative association between the abundance of bacitracin ARGs in the gut and gut microbiome Shannon index (Table S13). These findings indicate that greater gut microbiome diversity may be linked to a less abundant resistome. Further study is warranted to verify this relationship with a larger sample size at this sequencing depth.

Overall, there were few significant associations between environmental parameters and ARG abundance (Fig. 5). However, we found that there may be a relationship between contamination of household floors and the gut resistome; according to the path analysis, household soil/dust contamination slightly influenced the abundance of ARGs in the gut (Table S11). It is possible that dirt floors may be a source of ARGs from soil. Behavioral data was not collected in this study, so it is not clear how or if infants or children interacted with dirt on the floors. However, further study should include more careful observation and cataloging of participant behaviors and interactions with the environment.

### *E. coli* are important ARG bacterial hosts in the infant and child gut resistome

The results of bacterial hosts of ARG in this study were anticipated for infant and child guts. *E. coli* was a large fraction of the infant and child gut microbiome, which may explain why they are the most abundant ARG host. In a prior study of infants 1 year old in Copenhagen, Denmark, *E. coli* was also the most abundant ARG host [21]. Similar to our results, this study identified the most abundant host family as *Enterobacteriaceae*, with similar top genera (*Escherichia*, *Klebsiella*, and *Enterobacter*) [21] (Fig. 6; Figure S14). It has been demonstrated that *E. coli* have a critical role in shaping the abundance and diversity of the infant resistome, potentially due to their role as ARG sources and hosts [74]. ARG bacterial hosts may differ with age group or geography, as a study of ARG bacterial hosts in adults from Cornell, NY, US identified similar major families as the present study, but the overall abundances were different [75]. *Enterobacteriaceae* have been identified as the most common and important ARG hosts in the human gut, animal feces, and environmental samples (indoor environments, soil, water, air), highlighting this family’s contributions to AR in the human gut and the environmental resistome [76, 77]. Further studying the overlap in the human, animal, and environmental resistome, including hosts, is a crucial step to further understanding of the sharing between these matrices and its role in AR.

## Conclusion

This study analyzed the factors that help structure the gut microbiome and resistome in early life from a One Health perspective. We found that household environments with greater animal fecal contamination are associated with greater gut microbiome diversity in children and infants, however there is also higher abundance of potential pathogens in the gut. The resistome analysis of child and infant guts identified decreased ARG abundance with age. Other factors that may relate to resistome abundance include contamination of household floors and microbiome diversity. This study used long-read sequencing technology and a bioinformatic workflow that allowed ARG and microbial host annotation. These results are valuable because they link the gut microbiome to the resistome. While there were limitations in this analysis, such as missing survey data, shallow sequencing depth, and cross-sectional sampling, this study marks progress in characterizing the gut microbiomes and resistomes of this sensitive age group in an under-studied region. Future study should more systematically collect host exposure data, such as antibiotic use, comorbidities, and logging daily activities. Longitudinal study would also be a valuable next step in understanding how the gut microbiome and resistome develop in rural environments. We also recommend sequencing at a greater depth than the Flongle flow cells allowed, to give a more complete picture of the resistome in the future. Understanding the effects of environmental and animal exposures in the development of the gut microbiome and resistome is important for untangling the complex web of factors that impact these communities. This may result in the ability to manipulate these factors to optimize human health.

### Supplementary Information

**Additional file 1.**

## Data Availability

Sequence data and metadata for the samples in this study are deposited at the National Center for Biotechnology Information (NCBI) Sequence Read Archive (SRA) under the accession number PRJNA894152 (https://www.ncbi.nlm.nih.gov/sra/PRJNA894152).
