# Supplementary material for: Household environment and animal fecal contamination are critical modifiers of the gut microbiome and resistome in young children from rural Nicaragua
Source: Microbiome. 2023 Sep 15;11:207. doi: 10.1186/s40168-023-01636-5 (PMC10503196; doi:10.1186/s40168-023-01636-5)
Supplement: Supplementary file 2 — Additional file 1. [file 40168_2023_1636_MOESM1_ESM.docx]

Supplementary information for:

**Household environment and animal fecal contamination are critical modifiers of the gut microbiome and resistome in young children from rural Nicaragua**

Molly Mills^a,b^, Seungjun Lee^c^, Barbara A. Piperata^d^, Rebecca Garabed^e^, Boseung Choi^f^, and Jiyoung Lee^a,b,g^ *

^a^Division of Environmental Health Sciences, College of Public Health, The Ohio State University, Columbus, OH, USA

^b^Environmental Sciences Graduate Program, The Ohio State University, Columbus, OH, USA

^c^Department of Food Science and Nutrition, College of Fisheries Science, Pukyong National University, Busan, Republic of Korea

^d^Department of Anthropology, The Ohio State University, Columbus, OH, USA

^e^Department of Veterinary Preventive Medicine, The Ohio State University, OH, USA

^f^Division of Big Data Science, Korea University, Sejong, Republic of Korea

^g^Department of Food Science & Technology, The Ohio State University, Columbus, OH, USA

*Corresponding author: Jiyoung Lee, lee.3598@osu.edu

406 Cunz Hall, 1841 Neil Avenue, Columbus, Ohio 43210, USA

Included in this file:

**Supplementary Methods**

Droplet Digital^TM^ PCR system and conditions

Ethanol precipitation protocol

16S rRNA gene sequencing methods

Long-read sequence data filtering and normalization

**Supplementary Results**

Viral results

Fungal community results

**Supplementary Tables** (Tables S1-S13)

**Supplementary Figures** (Figures S1-S16)

**References**

**Supplementary Methods**

*Droplet Digital^TM^ PCR system and conditions*

The marker genes for the pathogenic *Escherichia coli* (*stx1*, *stx2*), *Arcobacter* spp., *Campylobacter* (targeting the 16S rRNA gene of *C. jejuni*, *C. coli*, and *C. lari*), and *Salmonella* (*invA*), and the microbial source tracking (MST) markers for ruminant fecal (Rum2Bac), human fecal (HF183), and dog fecal (BacCan-UCD) were quantified using the Droplet Digital^TM^ PCR (ddPCR) system (Bio-Rad, Hercules, California). All target genes primer sets were identified from the literature (Table S1), and assays were optimized for ddPCR priorthe to quantification of environmental samples. The EvaGreen ddPCR system was used to quantify *Arcobacter* spp., *Campylobacter*, and *Salmonella*. Evagreen ddPCR followed the QX200^TM^ ddPCR^TM^ EvaGreen® Supermix protocol (Bio-rad, Hercules, CA, USA). The PCR mixture included 10μL X200^TM^ ddPCR^TM^ EvaGreen® Supermix (Bio-rad), 250nM of primers, 2μL of DNA template, and nuclease free water, to a total volume of 20µL. The PCR cycling conditions were an initial cycle at 95°C for 10min, 40 cycles repeating 95°C for 30s and 60°C for 1min, and one final cycle at 98°C for 10min. Samples were cooled to 4°C.

A probe-based ddPCR system was used to quantify pathogenic *E. coli*, *Rum2Bac*, *Hf183*, and *BacCan-UCD*. These assays followed the ddPCR^TM^ Supermix for Probes protocol (Bio-rad). The PCR mixture contained with 10 μL 2x ddPCR^TM^ Supermix for Probes (Bio-rad), 250nM of primers and probe, 2μL of DNA template, and nuclease free water to a total volume to 20μL. The PCR cycling conditions for pathogenic *E. coli* were an initial cycle at 95°C for 10min, 40 cycles repeating 95°C for 30s and 60°C for 1min, and one cycle at 98°C for 10min. Samples were cooled to 4°C. The PCR cycling conditions for all MST markers were an initial step at 95°C for 10min, 44 cycles repeating 94°C for 30s and 60°C for 1min, and a final step at 98°C for 10min. Samples were cooled to 4°C.

Droplets were generated for both probe-based and EvaGreen ddPCR prior to PCR using the QX200^TM^ Droplet Generator (Bio-rad) and the Droplet Generation Oil for Probes (Bio-rad) or the QX200^TM^ Droplet Generation Oil for EvaGreen (Bio-rad), depending on the assay. Target genes were quantified using the QX200^TM^ Droplet Reader and QuantaSoft Software (Bio-rad).

*Ethanol precipitation protocol*

Child and infant fecal DNA samples were cleaned and concentrated for MinION sequencing using a basic ethanol precipitation protocol. 0.1 volume of 3M sodium acetate and 2.5-3 volumes of cold ethanol were added of the total DNA volume and mixed. The samples were left to precipitate overnight at -20°C to maximize yield. Samples were then centrifuged at 4°C, 13000rpm for 30min. The pelleted DNA was washed twice with 0.5mL cold 75% ethanol, and centrifuged at 4°C, 13000rpm for 10min between each wash. The ethanol was then removed, and samples were centrifuged one final time to remove any trace ethanol. The pelleted DNA was then air-dried for ~10min to ensure no ethanol contamination and resuspended in 30-50µL nuclease-free water, depending on the original DNA concentration.

*16S rRNA gene sequencing methods*

Out of 26 chicken fecal samples, 25 were sufficient quality for 16S rRNA gene sequencing. Out of 39 household soil/dust samples, only 2 were sufficient quality for 16S rRNA gene sequencing. The V4-V5 region of the 16S rRNA gene was targeted and amplified using Earth Microbiome Project (EMP) primers (515F/806R). 16S rRNA gene sequence data were demultiplexed at the facility. Chicken and soil samples were processed separately using the comprehensive pipeline QIIME2 (Bolyen et al., 2019). Sequences were quality filtered and trimmed using DADA2 (Callahan et al., 2016) with cut points of 294 base pair (bp) for the forward reads and 270 bp for the reverse reads of the chicken samples. Cut points of 296 bp for the forward reads and 269 bp for the reverse reads were used for the soil samples. Taxonomy was assigned on the SILVA132 16S rRNA gene classifier (99%) (Quast et al., 2013).Taxonomy was assigned on the SILVA132 16S rRNA gene classifier (99%) (Quast et al., 2013).

*Long-read sequence data filtering and normalization*

Eukaryotic and viral reads were filtered from data prior to the diversity analysis. Taxonomy results were filtered at a Kingdom, retaining only the Bacteria and Archaea. Before the calculation of diversity indices, taxonomic reads were normalized to an even depth by scaling all libraries to the median library size. This method of normalization was selected to retain as much of the full dataset as possible for power in statistical testing; because of the wide variety in library sizes, there was no clear rarefying threshold (McMurdie & Holmes, 2014; Weiss et al., 2017). 5 infants and 7 children were excluded from the Flongle flow cell diversity analysis because of small libraries (<100 reads). One infant sample was excluded from the MinION flow cell analysis because of library size (<200 reads). The data presented are from the Flongle flow cell analysis, unless otherwise stated. A comparison of the MinION and Flongle flow cell sequencing measures and library sizes after normalization are summarized in the Supplementary information (Table S2), as well as a comparison of taxonomy results (Figure S2). While the Flongle flow cells have less sequencing depth than the MinION flow cells, resulting in smaller library sizes, the cost effectiveness allows for sequencing a greater number of samples.

For more appropriate comparison of microbial communities sequenced via the 16S rRNA gene (chicken fecal and household soil/dust) to the WGS results (infant and child fecal), all samples were normalized together in R for that portion of the analysis. This included scaling each library to the mean library size of all samples and re-calculation of alpha diversity indices (Shannon index and Pielou’s evenness).

**Supplementary Results**

*Viral results*

Identification of viruses was dependent on the sequencing depth, so viral counts were related to library size. This prohibited further analysis of trends in viral communities. However, viruses were detected in 8/26 infant gut samples and 10/31 child gut samples. 43 unique viruses were identified in the Flongle analysis. Overall, the most abundant virus detected was crAssphage (Figure S6).

*Fungal community results*

Fungal communities were not detectable in the Flongle flow cell analysis, due to the limited sequencing depth. However fungal reads were detectable in 22/23 samples of the subset sequenced on the MinION flow cell. The library size of fungal communities was dependent on sequencing depth, with a range of 1-707 reads, so diversity indices could not accurately be calculated. However, a description of the composition of the fungal communities identified in child and infant gut microbiomes can be found in the supplementary data (Figure S7-S8).

**Supplementary Tables**

**Table S1.** Primer information/references

| **Target** | **Marker gene name** | **Primers (5’ -> 3’)** | **References** |
| --- | --- | --- | --- |
| *Salmonella* spp. | *inv*A | F: GTGAAATAATCGCCACGTCGGGCAA  R: TCATCGCACCGTCAAAGGAACC | Hein et al., 2006 |
| *Campylobacter* spp. | - | F: CTGCTTAACACAAGTTGAGTAGG  R: TTCCTTAGGTACCGTCAGAA | Josefsen et al., 2004 |
| *Arcobacter* spp. | - | F: GTCGTGCCAAGAAAAGCCA  R: TTCGCTTGCGCTGACAT | González et al., 2010 |
| Pathogenic shiga toxin-producing *Escherichia coli* (STEC) | *stx1* | F: GACTGCAAAGACGTATGTAGATTCG  R: ATCTATCCCTCTGACATCAACTGC  P: [FAM]-TGAATGTCATTCGCTCTGCAATAGGTACTC-[BHQ] | Ibekwe et al., 2002 |
|  | *stx2* | F: ATTAACCACACCCCACCG  R: GTCATGGAAACCGTTGTCAC  P: [HEX]-CAGTTATTTTGCTGTGGATATACGAGGGCTTG-[BHQ] |  |
| Human-specific fecal bacteria | HF183 | F: ATCATGAGTTCACATGTCCG  R: CGTAGGAGTTTGGACCGTGT  P: [FAM]-TGAGAGGAAGGTCCCCCACATTGGA-[MGB] | Green et al., 2014 |
| Dog-specific fecal bacteria | BacCan-UCD | F: TTTTCAGCCCCGTTGTTTCG  R: TGAGCGGGCATGGTCATATT  P: [FAM]-AGTCTACGCGGGCGTACT-[MGB] | Kildare et al. 2007 |
| Ruminant-specific fecal bacteria | Rum2Bac | F: GGAGCGCAGACGGGTTTT  R: AATCGGAGTTCCTCGTGATATCTA  P: [FAM]-TGGTGTAGCGGTGAAA-[MGB] | Mieszkin et al., 2010 |

**Table S2.** Comparison of annotation of antibiotic resistance genes (ARGs) between Resfinder, which only annotated acquired ARGs at a 90% sequence identity and 60% minimum length, and NanoARG, which was used in this analysis, and has more permissive parameters (E-value 1e−5, identity 25%, coverage 40%, --nk 15000). The total number of ARGs annotated in each sample (hits) by each method is shown in parenthesis.

| SampleID | Sample Type | Annotated ARGs (Resfinder) | Annotated ARGs (NanoARG) |
| --- | --- | --- | --- |
| B01 | Infant | *cfxA5* (Beta-lactam), *cfxA3* (Beta-lactam), *cfxA* (Beta-lactam), *cfxA4*(Beta-lactam) (4) | *bcrA, catQ, cfrC, CfxA2, CfxA6, mdtK, mexW, multidrug ABC transporter, OprZ, pgpB, rpoB2, tet40, transcriptional regulatory protein CpxR cpxR, ugd, vanH, vanR* (20) |
| B04 | Infant | ***erm*(F) (Macrolide), *erm*(F) (Macrolide), *tet*(Q) (Tetracycline), *tet*(X) (Tetracycline), *tet*(X) (Tetracycline)** (5) | *bacA, baeR,* ***ermF****, LlmA 23S ribosomal RNA methyltransferase, macB, major facilitator superfamily transporter, mdfA, mdtC, mefA, mexF, MIR, multidrug ABC transporter, PBP-1A, TaeA, tetA****, tetQ****,* ***tetX****, truncated putative response regulator ArlR, ugd, vanD, vanR* (28) |
| B08 | Infant | ***Tet*(Q) (Tetracycline), *cfxA6* (Beta-lactam)** (2) | *bacA, bcrA, bpeF, cdeA,CfxA2,* ***CfxA6,*** *class C, cystathionine beta-lyase patB, efrA, efrB, LlmA 23S ribosomal RNA methyltransferase, lsa, MCR-4, mdtE, mdtG, mexF, ompR, patA, patB, PmrF, rosA, rpoB2, Staphylococcus mupA conferring resistance to mupirocin, TaeA, tetA(48), tetB(60), tetM,* ***tetQ****, tetR, transcriptional regulatory protein CpxR cpxR, truncated putative response regulator ArlR, ugd, vanD, vanH, vanR, vanS, ykkC* (73) |
| B09 | Infant | None | *baeR, bcrA, mtrA, rosA, Staphylococcus mupA conferring resistance to mupirocin, truncated putative response regulator ArlR, ugd, vanE, vanH, vanR, vanRI, vanYG1* (13) |
| B13 | Infant | ***erm*(F) (Macrolide), *erm*(F) (Macrolide), *erm*(F) (Macrolide), *erm*(F) (Macrolide), *erm*(F) (Macrolide)** (5) | ***ermF****, kasugamycin resistance protein ksgA, macB, mdtE, tetA(48), transcriptional regulatory protein CpxR cpxR* (7) |
| B13b | Infant | None | *Kasugamycin resistance protein ksgA, mdtG, tetQ* (3) |
| B15 | Infant | None | None |
| B19 | Infant | ***Erm*(F) (Macrolide),** ***cfxA6* (Beta-lactam), *cfxA6* (Beta-lactam)** (3) | *adeH, bacA, bcrA,* ***CfxA6,*** *cystathionine beta-lyase patB, dfrA12, dfrA20, emrB, EmrB-QacA family major facilitator transporter,* ***ermF,*** *Escherichia coli mipA, kdpE, LlmA 23S ribosomal RNA methyltransferase, macA, macB, major facilitator superfamily transporter, mdtK, mdtO, mecC, mefE, mepA, mexB, mexD, mexF, NmcR, ompR, OXA, PBP-2X, penA, pgpB, PmrF, rosA, rpoB2, Staphylococcus mupA conferring resistance to mupirocin, tetA(48), tetQ, transcriptional regulatory protein CpxR cpxR, truncated putative response regulator ArlR, ugd, vanR, vanTrL, vatE* (58) |
| B20 | Infant | ***aph(6)-Id* (Aminoglycoside),** *aph(3'')-Ib* (Aminoglycoside), ***catQ* (Phenicol)** (2) | ***aph(6)-I,*** *bacA, bacterial regulatory protein LuxR, cat chloramphenicol acetyltransferase****, catQ***  *chloramphenicol exporter, dfrA25, DNA-binding protein H-NS, major facilitator superfamily transporter, mdtB, mdtG, mel, mexF, ompF, OprA, pgpB, rpoB2, sul2, tetA, ugd, vanR, vgaB* (24) |
| B21 | Infant | *aadA2* (Aminoglycoside), ***dfrA12* (Trimethoprim),** *qacE* (Disinfectanct), ***tet*(O) (Tetracycline)** (4) | *aadA, bacterial regulatory protein LuxR, bcrA,* ***dfrA12,*** *efrA, EmrB-QacA family major facilitator transporter, macA, marA, mdtE, mdtF, mdtP, ompR, patA, penA, rosB, Staphylococcus mupA conferring resistance to mupirocin, sul1, tet34,* ***tetO,*** *TolC, transcriptional regulatory protein CpxR cpxR, vanR* (23) |
| B24 | Infant | ***cfxA6* (Beta-lactam), *cfxA6* (Beta-lactam), *cfxA6* (Beta-lactam), *cfxA6* (Beta-lactam),** ***aph(6)-Id* (Aminoglycoside), *aph(6)-Id* (Aminoglycoside), *tet*(Q) (Tetracycline), *tet*(W) (Tetracycline), *tet*(Q) (Tetracycline), *tet*(Q) (Tetracycline)** (10) | *acrB, aph(3’’)-I,* ***aph(6)-I,*** *bacA, baeR, cat chloramphenicol acetyltransferase,* ***CfxA6****, class C, cmeB, cpxA, cystathionine beta-lyase patB, dfrK, efrA, emrA, emrB, emrR, Escherichia coli LamB, lmrD, lnuD, lsa, macB, mefA, mepA, mexH, ompR, oprN, optrA, patB, PBP-1A, rosA, rpoB2, sul2,* ***tetQ, tetW****, transcriptional regulatory protein CpxR cpxR, ugd, vanR, vanS, VatI* (65) |
| B25 | Infant | None | None |
| B26 | Infant | None | *acrB, adeC, bacA, baeR, CfxA6, class C, cob(I)alamin adenolsyltransferase, DNA-binding protein H-NS, ermF, kasugamycin resistance protein ksgA, mdtM, mdtO, mdtP, omp36, SAT-4, Serratia marcescens Omp1, tetO, TolC, ugd, vanR* (21) |
| B27 | Infant | ***Tet*(W) (Tetracycline)** (1) | *acrB, baeR, bcrC, cfrC, cmeB, cystathionine beta-lyase patB, ermF, LlmA 23S ribosomal RNA methyltransferase, macA, mdtF, mdtK, mexF, omp36, ompF, PBP-2X, rosA, Staphylococcus mupA conferring resistance to mupirocin, tetQ,* ***tetW,*** *vanR* (35) |
| B29 | Infant | None | *Escherichia coli LamB* (1) |
| B30 | Infant | ***cfxA6* (Beta-lactam**) (1) | ***CfxA6****, lnuD, vanR* (3) |
| B33 | Infant | *cfxA5* (Beta-lactam) (1) | *AcrF, ceoB, CfxA2, cystathionine beta-lyase patB, mdtA, mdtN, mdtO, mdtP, rpoB2, TaeA, transcriptional regulatory protein CpxR cpxR, ugd, vanD, vanR, vanRI* (17) |
| B34 | Infant | None | None |
| B38 | Infant | *blaTEM-1B* (Beta-lactam), *blaACI-1* (Beta-lactam), *blaTEM-122* (Beta-lactam), *blaTEM-104* (Beta-lactam) (4) | *adeB, eptA, mdtG, mdtP, Mrx, ompF, ompR, oqxA, oqxB, PBP-1A, penA, TEM, tetA(48), tetA(60), vanL* (17) |
| B39 | Infant | None | *efrB, Escherichia coli mipA, mtrA, OprA, truncated putative response regulator ArlR, ugd* (6) |
| B40 | Infant | ***tet*(Q) (Tetracycline), *tet*(40) (Tetracycline), *tet*(40) (Tetracycline)** (3) | *CfxA2, cob(I)alamin adenolsyltransferase, macB, mexA, mexF,* ***tet(40), tetQ,*** *ugd, ykkD* (10) |
| B41 | Infant | None | None |
| B45 | Infant | *cepA* (Beta-lactam), ***tet*(W) (Tetracycline),** ***erm*(X) (Macrolide), *erm*(X) (Macrolide), *erm*(X) (Macrolide), *erm*(X) (Macrolide), *erm*(X) (Macrolide), *erm*(X) (Macrolide)** (8) | *amrB, bcrA, class A,* ***ermX,***  *LlmA 23S ribosomal RNA methyltransferase, mepA, omp36, rosA,* ***tetW,*** *vanR, vanS* (13) |
| B46 | Infant | None | *bacA, kdpE, mexF, patA, rpoB2* (5) |
| B47 | Infant | *tet(O/W/O)-2* (Tetracycline), *tet(O/W)-2* (Tetracycline), *tet(O/W/O)-2* (Tetracycline) (3) | *acrA, acrB, adeR, aph(3’’’)-III, aph(6)-I, CfxA2, mepA, ompR, patA, patB, rosA, rosB, rpoB2, Staphylococcus mupA conferring resistance to mupirocin, tetW, TolC, ugd, vanTG, YojI* (23) |
| B49 | Infant | ***tet*(Q) (Tetracycline), *tet*(W) (Tetracycline)** (2) | *efrB, kdpE, lnuE, macA, mdtP, mexW, patA, penA, rpoB2, salA, tet40, tetB(60), tetM,* ***tetQ, tetW****, vanR, vanS* (18) |
| C01 | Child | ***Erm*(F) (Macrolide), *erm*(F) (Macrolide)** (2) | *Aac(6’)-I, adeJ, bcrA, CfxA2, CfxA6, cystathionine beta-lyase patB, DNA-binding transcriptional regulator gadX, EmrB-QacA family major facilitator transporter,* ***ermF,*** *gadW, LlmA 23S ribosomal RNA methyltransferase, lmrD, macB, mdtE, mdtF, mepA, mexA, PmrF, rosA, rpoB2, tetO, ugd, vanR, vgaE* (28) |
| C02 | Child | ***Erm*(F) (Macrolide), *erm*(F) (Macrolide)** (2) | *bacA, baeR, ceoB, cystathionine beta-lyase patB, DNA-binding protein H-NS, EmrB-QacA family major facilitator transporter,* ***ermF,*** *kdpE, MCR-4, mdtA, mdtB, mdtG, mdtK, mexX, multidrug ABC transporter, ompR, patB, PmrF, rpoB2, tet32, tetM, ugd, vanD* (30) |
| C06 | Child | None | *ompR, rosA, salA, tetO, ugd, vanH, vanR* (7) |
| C07 | Child | ***tet*(Q) (Tetracycline), *tet*(Q) (Tetracycline), *tet*(Q) (Tetracycline), *tet*(Q) (Tetracycline)**, ***cfxA6* (Beta-lactam), *cfxA5* (Beta-lactam), *cfxA6* (Beta-lactam),** *cfiA4* (Beta-lactam), *cfxA5* (Beta-lactam) (9) | *adeS, amrB, bcrA, carA, ccrA, CfxA2,* ***CfxA6,*** *cmeB, efrA, macB, mdsB, mexE, multidrug ABC transporter, PmrF, rosA, rpoB2, tet35,* ***tetQ,*** *truncated putative response regulator ArlR, ugd, vanH, vanR, vanTG* (46) |
| C08 | Child | None | *acrA, acrB, adeR, cdeA, cystathionine beta-lyase patB, dfrG, kasugamycin resistance protein ksgA, kdpE, macB, mexX, multidrug ABC transporter, rosA, smeR, tetQ, transcriptional regulatory protein CpxR cpxR, vanB, vanE, vanR* (22) |
| B10 | Child | ***Sul2* (Sulfonamide)** (1) | *Aph(3’’)-I, aph(6)-I, bcrA, cAMP-regulatory protein, cat chloramphenicol acetyltransferase, class A, class C, cmeB, efrB, emrK, Escherichia coli LamB, kasugamycin resistance protein ksgA, lsa, macB, mexF, oprN, patB, rpoB2,* ***sul2****, tetW, transcriptional regulatory protein CpxR cpxR, truncated putative response regulator ArlR, vanR, vanS, ykkC* (31) |
| C13 | Child | ***cfxA6* (Beta-lactam), *tet*(40) (Tetracycline)** (2) | *bcrA, cdeA,* ***CfxA6,*** *cystathionine beta-lyase patB, patB, rpoB2, Staphylococcus mupA conferring resistance to mupirocin,* ***tet40****, tetM, tetQ, tetW* (15) |
| C14 | Child | ***Tet*(O) (Tetracycline)** (1) | *CfxA6, efrA, tetM,* ***tetO,*** *truncated putative response regulator ArlR* (5) |
| C15 | Child | None | *Cystathionine beta-lyase patB, efrA* (2) |
| C16 | Child | ***Tet*(W) (Tetracycline)** (1) | *adeB, bcrA, ermG, rosA,* ***tetW*** (5) |
| B17 | Child | None | *acrB, bcrA, cdeA, efrA, kdpE, ompR, patA, PmrF, ramA, rpoB2, smeR, Staphylococcus mupA conferring resistance to mupirocin, truncated putative response regulator ArlR, ugd, vanR, vgaE* (21) |
| C20 | Child | None | *Multidrug ABC transporter, rpoB2, ugd* (3) |
| C21 | Child | None | *bacA, multidrug ABC transporter, RlmA(II), rosA, TaeA, truncated putative response regulator ArlR, ugd, vanH, vanRI, vanTmL* (12) |
| C23 | Child | None | *tlcC, truncated putative response regulator ArlR, ugd, vanR, ykkD* (5) |
| C24 | Child | None | *bacA, multidrug ABC transporter, RlmA(II), rosA, TaeA, truncated putative response regulator ArlR, ugd, vanH, vanRI, vanTmL* (13) |
| C25 | Child | *cfxA3* (Beta-lactam), *blaTEM-76* (Beta-lactam), *blaTEM-1A* (Beta-lactam), *blaTEM-112* (Beta-lactam), *blaTEM-54* (Beta-lactam), *blaTEM-141* (Beta-lactam) (6) | *baeR, CfxA2, cpxA, cystathionine beta-lyase patB, LlmA 23S ribosomal RNA methyltransferase, mexX, rosA, Serratia marcescens Omp1, TEM, tet34, ugd* (13) |
| C26 | Child | ***cfxA6* (Beta-lactam), *cfxA6* (Beta-lactam)** (2) | *adeJ, bacA, bacterial regulatory protein LuxR, baeR, bcrA, CfxA2,* ***CfxA6,*** *cmeB, cystathionine beta-lyase patB, dfrF, efrB, LlmA 23S ribosomal RNA methyltransferase, mepA, mexF, ompR, patB, PmrF, rpoB2, Staphylococcus mupA conferring resistance to mupirocin, tetA(48), tetM, tetQ, ugd, vanE* (32) |
| C27 | Child | ***tet*(Q) (Tetracycline), *tet*(O) (Tetracycline),** *tet*(O/32/O) (Tetracycline) (3) | *aadE, bcrA, cdeA, efrB, ermF, LlmA 23S ribosomal RNA methyltransferase, lnuA, mexF, mtrA, ompR, TaeA, tet34, tetA(48), tetM,* ***tetO, tetQ,*** *tetW, truncated putative response regulator ArlR, ugd, vanR* (30) |
| C28 | Child | *tet*(Q) (Tetracycline) (1) | *tetQ*, *rpoB2*, *bcrA*, *CfxA2* (4) |
| C29 | Child | *tet*(Q) (Tetracycline) (1) | *Escherichia coli LamB* (1) |
| C30 | Child | None | None |
| C31 | Child | *cfxA4* (Beta-lactam), ***erm*(F) (Macrolide)** (2) | *bacA, bcrA, carA, CfxA2, cystathionine beta-lyase patB, efrA, efrB,* ***ermF,*** *LlmA 23S ribosomal RNA methyltransferase, mepA, ompR, poxtA, smeR, Staphylococcus mupA conferring resistance to mupirocin, tetA(48), tetP, transcriptional regulatory protein CpxR cpxR, ugd, vanH, vanR, vanTG, vatB* (39) |
| C33 | Child | ***Tet*(Q) (Tetracycline), *cfxA6* (Beta-lactam), *erm*(F) (Macrolide)** (3) | ***CfxA6,*** *cmeB, cystathionine beta-lyase patB, efrB,* ***ermF,*** *macA, macB, MCR-3, mdtK, mexB, multidrug ABC transporter, pgpB, Staphylococcus mupA conferring resistance to mupirocin,* ***tetQ,*** *tetT, transcriptional regulatory protein CpxR cpxR, truncated putative response regulator ArlR, vanA, vanH, vanR, vanS* (26) |
| C34 | Child | None | *bcrA, efrB, LlmA 23S ribosomal RNA methyltransferase, mexB, mexF, multidrug ABC transporter, TEM, tet44, truncated putative response regulator ArlR, ugd, vanD, vanE, vanG, vanR, vanRI, vanTG, vanY* (23) |
| C35 | Child | *Erm*(F) (Macrolide), ***tet*(Q) (Tetracycline), *tet*(Q) (Tetracycline), *tet*(Q) (Tetracycline)** (4) | *bacA, bcrA, efrA, efrB, ermG, LlmA 23S ribosomal RNA methyltransferase, macB, mepA, ompR, patB, PBP-1B, penA, PmrF, rosA, rpoB2, smeF, tetA(48),* ***tetQ,*** *tetW, truncated putative response regulator ArlR, ugd, vanD, vanRI, vanTG* (34) |
| C36 | Child | ***cfxA6* (Beta-lactam)** (1) | *bacA,* ***CfxA6,*** *mdtE, mdtF, multidrug ABC transporter, ugd, vanR, vanR* (9) |
| C41 | Child | None | *LlmA 23S ribosomal RNA methyltransferase, ompR, truncated putative response regulator ArlR, vanD, vanE, vanY* (7) |
| C42 | Child | ***cfxA6* (Beta-lactam)** (1) | ***CfxA6,*** *multidrug ABC transporter, otr(B), PmrF, rosA, rpoB2, smeE, tetQ, ugd, vanH, vanR, vanRI* (18) |
| B42 | Child | ***Aph(3’’)-Ib* (Aminoglycoside)** (1) | ***Aph(3’’)-I,*** *arlS, bcrA, dfrA3, efrA, efrB, EmrB-QacA family major facilitator transporter, mexA, mtrA, PBP-2X, PmrF, rpoB2, sul2, tetM, tetO, tetS, transcriptional regulatory protein CpxR cpxR, truncated putative response regulator ArlR, ugd, vanH, vanR, vgaB* (26) |
| C48 | Child | None | None |
| C51 | Child | None | *bcrA, catV, class A, dfrA1, efrB, ermF, macB, MCR-3, mepA, mexF, mtrA, ompR, tetQ, ugd, vanR, vanRI* (22) |

**Table S3.** A comparison of Oxford Nanopore Technologies’ Flongle flow cells and MinION flow cells in MinION long read sequencing.

| Sequencing Measures | Flongle flow cell | MinION flow cell |
| --- | --- | --- |
| Average reads generated/sample | 3,078 | 26,758 |
| Average reads classified/sample | 1,018 | 8,908 |
| Average reads generated/sequencing run | 49,292 | 339,990 |
| Average reads classified/sequencing run | 13,601 | 98,944 |
| Average quality score | 9.22 | 11.2 |
| Average read length (bp) | 1,733 | 3,268 |
| Median library size/sample after normalizing (bacteria & archaea) | 756 | 4,885 |

**Table S4.** Alpha diversity indices of all child and infant fecal DNA samples. Samples that were excluded from the diversity analysis for low library sizes are italicized and highlighted in grey.

| **Sample** | **Household** | **Type** | **Shannon index** | **Evenness** |
| --- | --- | --- | --- | --- |
| C01 | 1 | Child | 4.74 | 0.86 |
| C02 | 2 | Child | 4.53 | 0.83 |
| C06 | 6 | Child | 3.04 | 0.68 |
| C07 | 7 | Child | 4.74 | 0.84 |
| C08 | 8 | Child | 4.72 | 0.82 |
| B10 | 10 | Child | 3.03 | 0.72 |
| C13 | 13 | Child | 4.73 | 0.86 |
| C14 | 14 | Child | 4.09 | 0.93 |
| C15 | 15 | Child | 3.93 | 0.95 |
| *C16* | *16* | *Child* | *NA* | *NA* |
| B17 | 17 | Child | 4.31 | 0.81 |
| *C20* | *20* | *Child* | *NA* | *NA* |
| C21 | 21 | Child | 4.50 | 0.85 |
| C23 | 23 | Child | 4.40 | 0.93 |
| *C24* | *24* | *Child* | *NA* | *NA* |
| C25 | 25 | Child | 4.71 | 0.85 |
| C26 | 26 | Child | 4.93 | 0.84 |
| C27 | 27 | Child | 4.20 | 0.78 |
| C28 | 28 | Child | 4.33 | 0.89 |
| *C29* | *29* | *Child* | *NA* | *NA* |
| *C30* | *30* | *Child* | *NA* | *NA* |
| C31 | 31 | Child | 4.95 | 0.87 |
| C33 | 33 | Child | 4.70 | 0.89 |
| C34 | 34 | Child | 4.97 | 0.86 |
| C35 | 35 | Child | 4.66 | 0.85 |
| C36 | 36 | Child | 3.90 | 0.82 |
| *C41* | *41* | *Child* | *NA* | *NA* |
| C42 | 42 | Child | 4.50 | 0.82 |
| B42 | 42 | Child | 3.19 | 0.70 |
| *C48* | *48* | *Child* | *NA* | *NA* |
| C51 | 51 | Child | 4.02 | 0.85 |
| B01 | 1 | Infant | 4.06 | 0.80 |
| B04 | 4 | Infant | 3.32 | 0.76 |
| B08 | 8 | Infant | 4.12 | 0.84 |
| B09 | 9 | Infant | 3.97 | 0.79 |
| B13 | 13 | Infant | 3.09 | 0.72 |
| B13b | 13 | Infant | 4.06 | 0.85 |
| *B15* | *15* | *Infant* | *NA* | *NA* |
| B19 | 19 | Infant | 3.33 | 0.75 |
| B20 | 20 | Infant | 3.69 | 0.72 |
| B21 | 21 | Infant | 3.42 | 0.76 |
| B24 | 24 | Infant | 3.76 | 0.83 |
| *B25* | *25* | *Infant* | *NA* | *NA* |
| B26 | 26 | Infant | 3.43 | 0.70 |
| B27 | 27 | Infant | 4.08 | 0.76 |
| B29 | 29 | Infant | 2.65 | 0.56 |
| *B30* | *30* | *Infant* | *NA* | *NA* |
| B33 | 33 | Infant | 4.57 | 0.89 |
| *B34* | *34* | *Infant* | *NA* | *NA* |
| B38 | 38 | Infant | 3.22 | 0.70 |
| B39 | 39 | Infant | 2.87 | 0.67 |
| B40 | 40 | Infant | 3.14 | 0.70 |
| *B41* | *41* | *Infant* | *NA* | *NA* |
| B45 | 45 | Infant | 3.70 | 0.76 |
| B46 | 46 | Infant | 3.29 | 0.78 |
| B47 | 47 | Infant | 3.83 | 0.74 |
| B49 | 49 | Infant | 4.08 | 0.79 |

**Table S5.** The 10 most abundant ARGs and their antibiotic class in all fecal samples, infants, and children. The percent shown is the percent of the total ARGs.

|  | **All** | | | **Infant** | | | **Child** | | |
| --- | --- | --- | --- | --- | --- | --- | --- | --- | --- |
|  | ARG | Antibiotic Class | % | ARG | Antibiotic Class | % | ARG | Antibiotic Class | % |
| **1** | *vanR* | Glycopeptide | 6.0 | *mdtN* | Multidrug | 6.7 | *ugd* | Peptide | 7.1 |
| **2** | *ugd* | Peptide | 5.0 | *tet34* | Tetracycline | 6.7 | *vanR* | Glycopeptide | 6.6 |
| **3** | *tet34* | Tetracycline | 3.6 | *vanR* | Glycopeptide | 5.4 | *ArlR* | Unclassified | 5.5 |
| **4** | *rpoB2* | Multidrug | 3.5 | *ermF* | MLS | 5.1 | *bcrA* | Bacitracin | 4.8 |
| **5** | *mdtN* | Multidrug | 3.2 | *ugd* | Peptide | 3.0 | *ompR* | Multidrug | 3.7 |
| **6** | *ArlR* | Unclassified | 3.2 | *rpoB2* | Multidrug | 2.9 | *rpoB2* | Multidrug | 4.2 |
| **7** | *ermF* | MLS | 3.2 | *CfxA6* | Beta-lactam | 2.6 | *rosA* | Fosmidomycin | 3.4 |
| **8** | *bcrA* | Bacitracin | 2.9 | *mexF* | Multidrug | 2.3 | *tetQ* | Tetracycline | 3.2 |
| **9** | *ompR* | Multidrug | 2.8 | *tetQ* | Tetracycline | 2.0 | *ABC transporter* | Multidrug | 2.9 |
| **10** | *tetQ* | Tetracycline | 2.6 | *Escherichia coli LamB* | Multidrug | 1.7 | *CfxA6* | Beta-lactam | 2.5 |

**Table S6.** The 10 most abundant genera in each household floor dust/soil sample are shown as a relative abundance of the total microbial community (%). There was a large difference in the depth of each library, in addition to differences in the microbial communities’ compositions. The sample S19 was from household #19 and was a composite sample from dirt floors (all dirt floors in the house). The sample S33 was from household #33. The sample was a composite of the dirt floor of the kitchen and the cement floors in the living room and bedrooms.

|  | **S19** | | | **S33** | | |
| --- | --- | --- | --- | --- | --- | --- |
|  | Genus | Phyla | % | Genus | Phyla | % |
| 1 | *Halomonas* | Proteobacteria | 0.091 | *Nesterenkonia* | Actinobacteria | 0.167 |
| 2 | *Nesterenkonia* | Actinobacteria | 0.045 | *Brachybacterium* | Actinobacteria | 0.134 |
| 3 | *Aliidiomarina* | Proteobacteria | 0.041 | *Glutamicibacter* | Actinobacteria | 0.078 |
| 4 | *Pseudomonas* | Proteobacteria | 0.033 | *Zhihengliuella* | Actinobacteria | 0.064 |
| 5 | *Bacteroides* | Bacteroidetes | 0.029 | *Ornithinimicrobium* | Actinobacteria | 0.054 |
| 6 | *Salinimicrobium* | Bacteroidetes | 0.021 | *Corynebacterium* | Actinobacteria | 0.051 |
| 7 | *Marinimicrobium* | Proteobacteria | 0.016 | *Ornithinicoccus* | Actinobacteria | 0.049 |
| 8 | *Marinobacter* | Proteobacteria | 0.016 | *Dietzia* | Actinobacteria | 0.041 |
| 9 | *Nocardioides* | Actinobacteria | 0.016 | *Nocardioides* | Actinobacteria | 0.037 |
| 10 | *Bacillus* | Firmicutes | 0.015 | *Brevibacterium* | Actinobacteria | 0.033 |
| **Total OTUs** | **39545** | | | **5739** | | |

**Table S7.** Path analysis results for the response variable of Shannon index (gut microbiome diversity), the known explanatory variable of age, and the mediating variables of water score (total number of pathogens detected in the household water via ddPCR) and soil score (the total number of MST markers and pathogens detected in household soil/dust via ddPCR).

| **Path Analysis** | | | | | | | | | | |
| --- | --- | --- | --- | --- | --- | --- | --- | --- | --- | --- |
|  | | Estimate | | Std.Err | | z-value | P(>\|z\|) | | Std estimate | |
| WaterScore ~ |  |  | |  | |  |  | |  | |
| Age | (a1) | 0 | | 0.004 | | 0.112 | 0.911 | | 0.019 | |
| SoilScore ~ |  |  | |  | |  |  | |  | |
| Age | (a2) | 0.013 | | 0.010 | | 1.348 | 0.178 | | 0.222 | |
| Shannon ~ |  |  | |  | |  |  | |  | |
| Age | (c) | 0.016 | | 0.003 | | 4.691 | 0 | | 0.544 | |
| WaterScore | (b1) | 0.426 | | 0.125 | | 3.395 | 0.001 | | 0.271 | |
| SoilScore | (b2) | 0.133 | | 0.055 | | 2.412 | 0.016 | | 0.237 | |
| **Mediating effect** | | | | | | | | | | |
| Effect | Estimate | | Std.Err | | z-value | | | P(>\|z\|) | | Std estimate |
| Direct effect | 0.016 | | 0.003 | | 4.691 | | | 0 | | 0.544 |
| Indirect effect1 -water | 0 | | 0.002 | | 0.112 | | | 0.911 | | 0.007 |
| Indirect effect2 - soil | 0.002 | | 0.002 | | 1.177 | | | 0.239 | | 0.061 |
| Total effect | 0.018 | | 0.004 | | 4.437 | | | 0 | | 0.6 |

**Table S8.** Path analysis results for the response variable of Shannon index (gut microbiome diversity), the known explanatory variable of age, and the mediating variables of environmental score (total number of pathogens detected in the household water and household soil/dust and total number of MST markers detected in household soil/dust via ddPCR).

| **Path Analysis** | | | | | | | | | | |
| --- | --- | --- | --- | --- | --- | --- | --- | --- | --- | --- |
|  |  | Estimate | | Std.Err | | z-value | | P(>\|z\|) | | Std estimate |
| EnvScore ~ |  |  | |  | |  | |  | |  |
| Age | (a1) | 0.014 | | 0.009 | | 1.487 | | 0.137 | | 0.244 |
| Shannon ~ |  |  | |  | |  | |  | |  |
| Age | (c) | 0.016 | | 0.004 | | 4.335 | | 0 | | 0.551 |
| EnvScore | (b1) | 0.145 | | 0.063 | | 2.305 | | 0.021 | | 0.293 |
| **Mediating effect** | | | | | | | | | | |
| Effect | Estimate | | Std.Err | | z-value | | P(>\|z\|) | | Std estimate | |
| Direct effect | 0.016 | | 0.004 | | 4.335 | | 0 | | 0.551 | |
| Indirect effect | 0.002 | | 0.002 | | 1.250 | | 0.211 | | 0.071 | |
| Total effect | 0.018 | | 0.004 | | 4.705 | | 0 | | 0.018 | |

**Table S9.** Path analysis results for the response variable of Shannon index (gut microbiome diversity), the known explanatory variable of delivery mode (vaginal v. cesarean), and the mediating variables of water score (total number of pathogens detected in the household water via ddPCR) and soil score (the total number of MST markers and pathogens detected in household soil/dust via ddPCR).

| **Path Analysis** | | | | | | | | | |
| --- | --- | --- | --- | --- | --- | --- | --- | --- | --- |
|  | | Estimate | | Std.Err | | z-value | P(>\|z\|) | | Std estimate |
| WaterScore ~ |  |  | |  | |  |  | |  |
| Type_birth | (a1) | 0.228 | | 0.198 | | 1.148 | 0.251 | | 0.185 |
| SoilScore ~ |  |  | |  | |  |  | |  |
| Type_birth | (a2) | 0.715 | | 0.456 | | 1.567 | 0.117 | | 0.25 |
| Shannon ~ |  |  | |  | |  |  | |  |
| Type_birth | (c) | 0.168 | | 0.197 | | 0.852 | 0.394 | | 0.119 |
| WaterScore | (b1) | 0.443 | | 0.156 | | 2.841 | 0.005 | | 0.385 |
| SoilScore | (b2) | 0.178 | | 0.068 | | 2.627 | 0.009 | | 0.361 |
| **Mediating effect** | | | | | | | | | |
| Effect | Estimate | | Std estimate | | P(>\|z\|) | | |  |  |
| Direct effect | 0.168 | | 0.119 | | 0.394 | | |  |  |
| Indirect effect1 -water | 0.101 | | 0.071 | | 0.287 | | |  |  |
| Indirect effect2 - soil | 0.127 | | 0.09 | | 0.178 | | |  |  |
| Total effect | 0.396 | | 0.281 | | 0.075 | | |  |  |

**Table S10.** Path analysis results for the response variable of Shannon index (gut microbiome diversity), the known explanatory variable of delivery mode (vaginal v. cesarean), and the mediating variables of environmental score (total number of pathogens detected in the household water and household soil/dust and total number of MST markers detected in household soil/dust via ddPCR).

| **Path Analysis** | | | | | | | | |
| --- | --- | --- | --- | --- | --- | --- | --- | --- |
|  |  | Estimate | Std.Err | | z-value | P(>\|z\|) | | Std estimate |
| EnvScore ~ |  |  |  | |  |  | |  |
| Type_birth | (a1) | 0.942 | 0.428 | | 2.199 | 0.028 | | 0.34 |
| Shannon ~ |  |  |  | |  |  | |  |
| Type_birth | (c) | 0.218 | 0.208 | | 1.052 | 0.293 | | 0.162 |
| EnvScore | (b1) | 0.189 | 0.075 | | 2.522 | 0.012 | | 0.389 |
| **Mediating effect** | | | | | | | | |
| Effect | Estimate | | | Std estimate | | | P(>\|z\|) | |
| Direct effect | 0.218 | | | 0.162 | | | 0.293 | |
| Indirect effect | 0.178 | | | 0.132 | | | 0.097 | |
| Total effect | 0.396 | | | 0.295 | | | 0.061 | |

**Table S11.** Path analysis results for the response variable of total ARG abundance (normalized ARG counts/gigabase pair), the known explanatory variable of age, and the mediating variables of water score (total number of pathogens detected in the household water via ddPCR) and soil score (the total number of MST markers and pathogens detected in household soil/dust via ddPCR).

| **Path Analysis** | | | | | | | | | | |
| --- | --- | --- | --- | --- | --- | --- | --- | --- | --- | --- |
|  | | Estimate | | Std.Err | | z-value | P(>\|z\|) | | Std estimate | |
| WaterScore ~ |  |  | |  | |  |  | |  | |
| Age | (a1) | -0.001 | | 0.004 | | -0.247 | 0.805 | | -0.04 | |
| SoilScore ~ |  |  | |  | |  |  | |  | |
| Age | (a2) | 0.013 | | 0.009 | | 1.353 | 0.176 | | 0.214 | |
| Total ARGs ~ |  |  | |  | |  |  | |  | |
| Age | (c) | -0.017 | | 0.006 | | -2.7 | 0.007 | | -0.405 | |
| WaterScore | (b1) | -0.037 | | 0.241 | | -0.152 | 0.879 | | -0.022 | |
| SoilScore | (b2) | 0.184 | | 0.108 | | 1.713 | 0.087 | | 0.257 | |
| **Mediating effect** | | | | | | | | | | |
| Effect | Estimate | | Std.Err | | z-value | | | P(>\|z\|) | | Std estimate |
| Direct effect | -0.017 | | 0.006 | | -2.700 | | | 0.007 | | -0.405 |
| Indirect effect1 -water | 0.000 | | 0.000 | | 0.129 | | | 0.897 | | 0.001 |
| Indirect effect2 - soil | 0.002 | | 0.002 | | 1.062 | | | 0.288 | | 0.055 |
| Total effect | -0.015 | | 0.006 | | -2.296 | | | 0.022 | | -0.349 |

**Table S12.** Path analysis results for the response variable of total ARG abundance (normalized ARG counts/gigabase pair), the known explanatory variable of age, and the mediating variables of environmental score (total number of pathogens detected in the household water and household soil/dust and total number of MST markers detected in household soil/dust via ddPCR).

| **Path Analysis** | | | | | | | | | | |
| --- | --- | --- | --- | --- | --- | --- | --- | --- | --- | --- |
|  |  | Estimate | | Std.Err | | z-value | | P(>\|z\|) | | Std estimate |
| EnvScore ~ |  |  | |  | |  | |  | |  |
| Age | (a1) | 0.012 | | 0.009 | | 1.313 | | 0.189 | | 0.208 |
| Total ARGs ~ |  |  | |  | |  | |  | |  |
| Age | (c) | -0.017 | | 0.006 | | -2.620 | | 0.009 | | -0.396 |
| EnvScore | (b1) | 0.173 | | 0.115 | | 1.502 | | 0.133 | | 0.227 |
| **Mediating effect** | | | | | | | | | | |
| Effect | Estimate | | Std.Err | | z-value | | P(>\|z\|) | | Std estimate | |
| Direct effect | -0.017 | | 0.006 | | -2.620 | | 0.009 | | -0.396 | |
| Indirect effect | 0.002 | | 0.002 | | 0.988 | | 0.323 | | 0.047 | |
| Total effect | -0.015 | | 0.006 | | -2.292 | | 0.022 | | -0.015 | |

**Table S13.** The results from Spearman’s rank correlation between the normalized ARG abundance and gut microbiome Shannon index and evenness. The resistome and microbiome data are from the MinION flow cell analysis (*n*=22). Correlations were computed for the total ARG abundance and the 5 most abundant ARG classes in this resistome data (multidrug, MLS, glycopeptide, tetracycline, and bacitracin). Both the overall *p* value for the correlation and the ρ value are shown. Significant correlations (*p*<0.05) are bolded and highlighted with a dashed box.

|  | Total ARG abundance | Multidrug ARG abundance | MLS ARG abundance | Glycopeptide ARG abundance | Tetracycline ARG abundance | Bacitracin ARG abundance |
| --- | --- | --- | --- | --- | --- | --- |
| Microbiome Shannon index | p = 0.11 | **p = 0.04** | p = 0.17 | p = 0.81 | p = 0.55 | **p = 0.029** |
|  | ρ = -0.35 | **ρ = -0.44** | ρ = -0.30 | ρ = 0.05 | ρ = 0.13 | **ρ = -0.23** |
| Microbiome evenness | p = 0.14 | **p = 0.04** | p = 0.67 | p = 0.29 | p = 0.51 | p = 0.11 |
|  | ρ = -0.32 | **ρ = -0.44** | ρ = -0.10 | ρ = -0.24 | ρ = 0.15 | ρ = -0.35 |


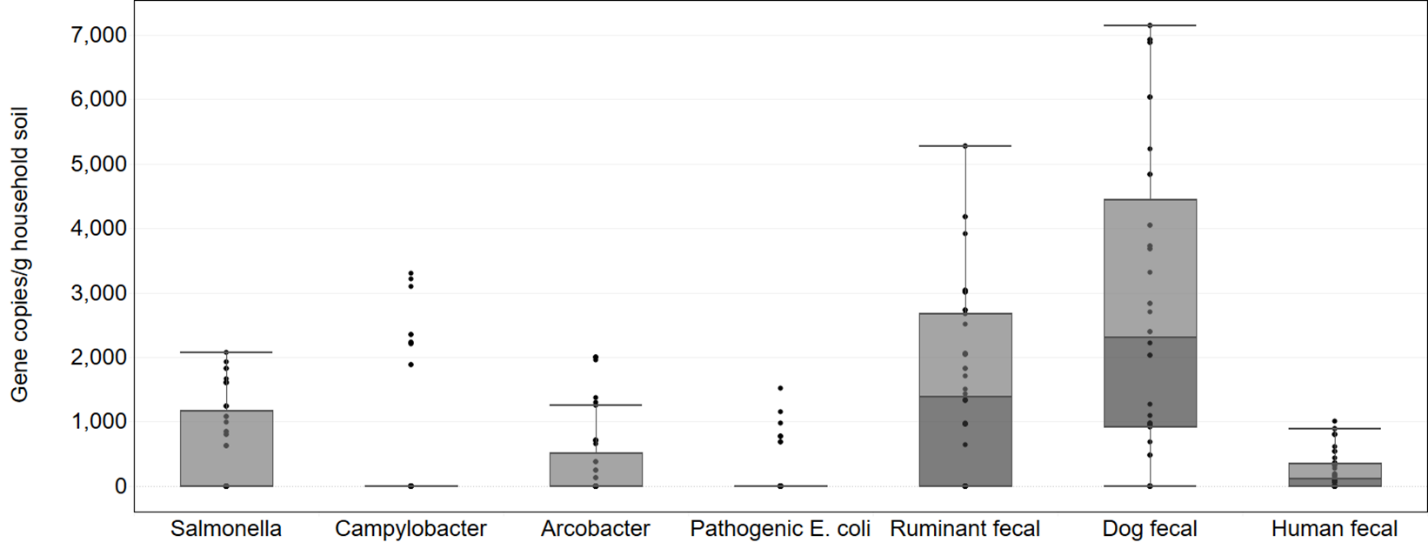
**Supplementary Figures**

**Figure S1.** The concentration of marker genes in gene copies/g of household soil/dust from all households. The marker genes include 4 enteric pathogens (*Salmonella*, *Campylobacter*, *Arcobacter*, pathogenic *Escherichia coli*), and 3 microbial source tracking (MST) markers for host-specific fecal bacteria (ruminant, dog, human).


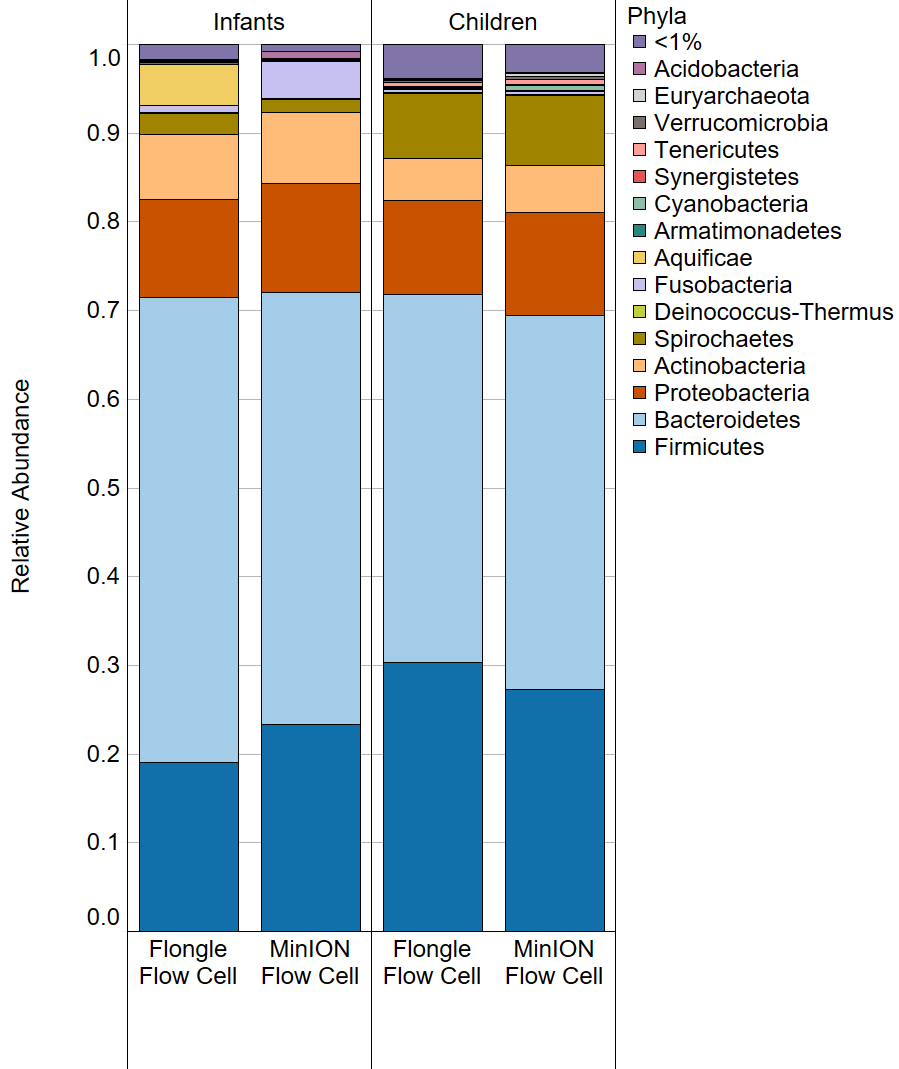


**Figure S2.** A comparison of the composition at a phyla level of the child and infant gut microbiomes sequenced using Flongle and MinION flow cells.


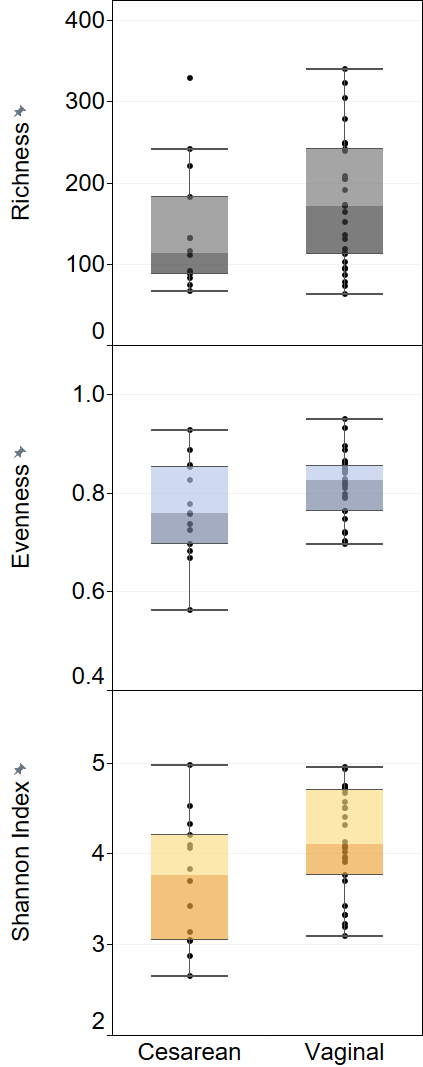


*p* = 0.10

*p* = 0.047*

(b)


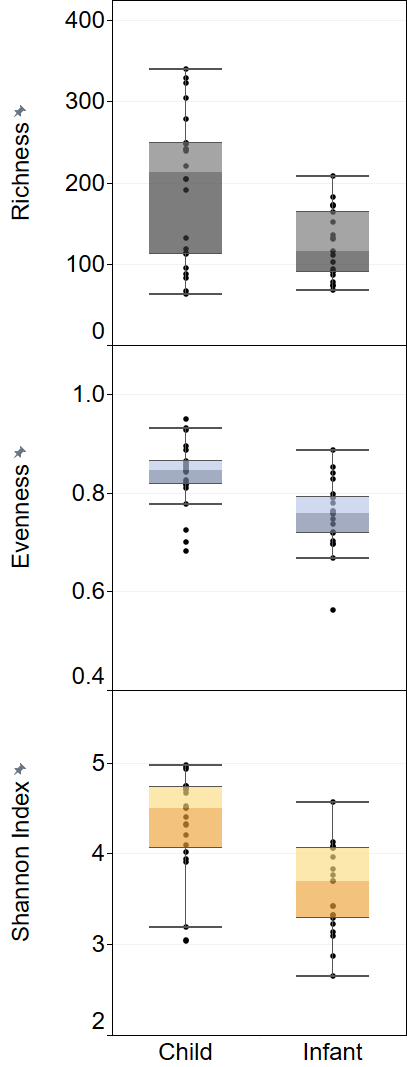


*p* = 9.4e-6***

*p* = 2.9e-5***

(a)

**Figure S3.** The comparison between alpha diversity indices is shown between children and infants (a) and by delivery mode (b) for child and infant fecal samples. Wilcoxon rank sum test results are shown for each comparison. **p*<0.05, ***p*<0.01, ****p*<0.001

**Figure S4.** Spearman correlation results for alpha diversity indices and numerical metadata categories. (a) and quantified microbial source tracking and pathogens via ddPCR; (b) in child and infant fecal samples. Ruminant fecal bacteria (Rum2Bac) correlation results are shown twice, with and without the 2 outliers removed. Correlation *p* and *rho* values are presented. **p*<0.05, ***p*<0.01, ****p*<0.001


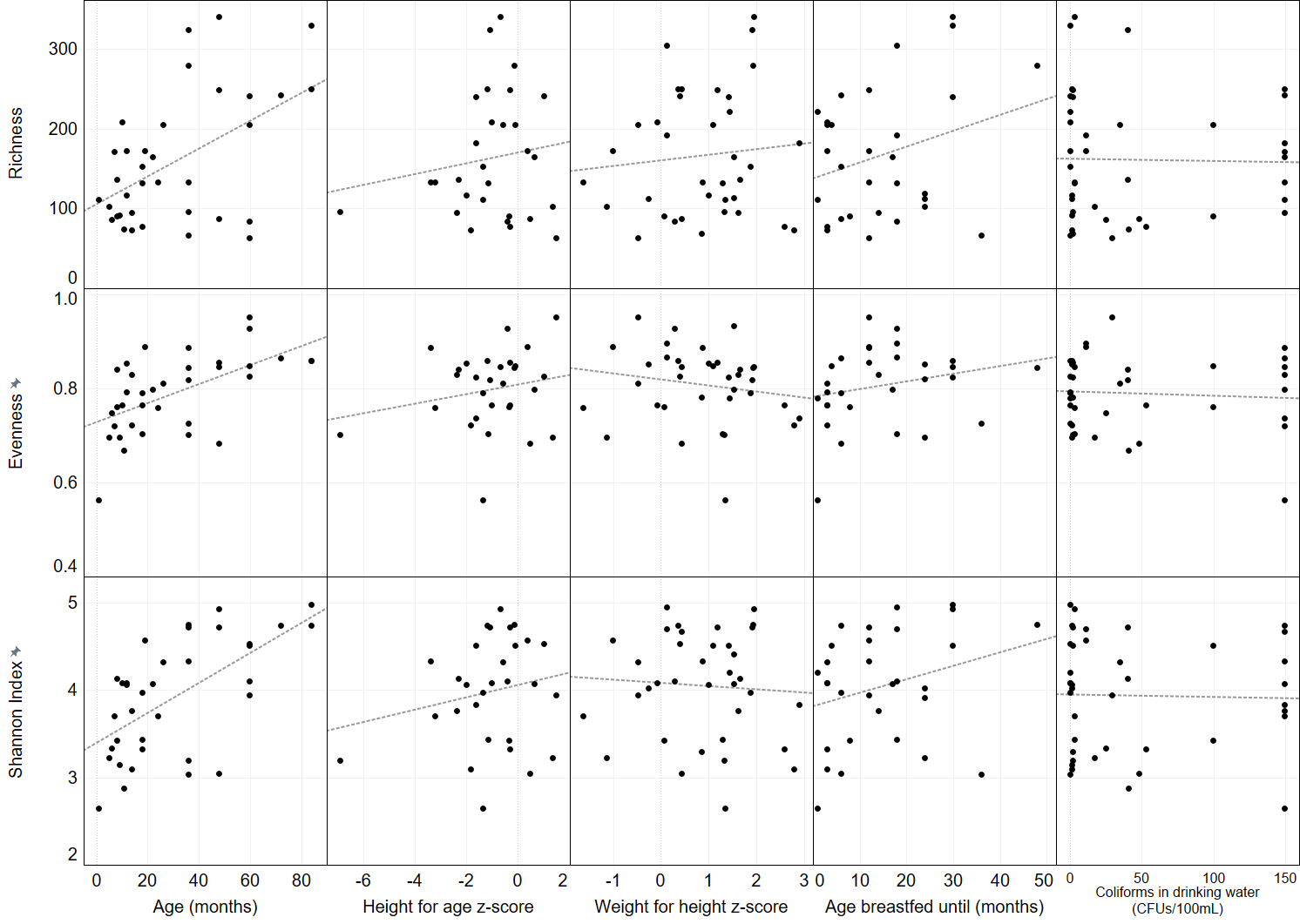


***

*p* = 4.8e-05

rho = 0.62

*p* = 6.3e-05

rho = 0.61

***

*p* > 0.05

*p* > 0.05

*p* > 0.05

*p* > 0.05

*p* = 0.13

rho = 0.28

*p* = 0.098

rho = 0.31

*p* > 0.05

*p* > 0.05

(a)


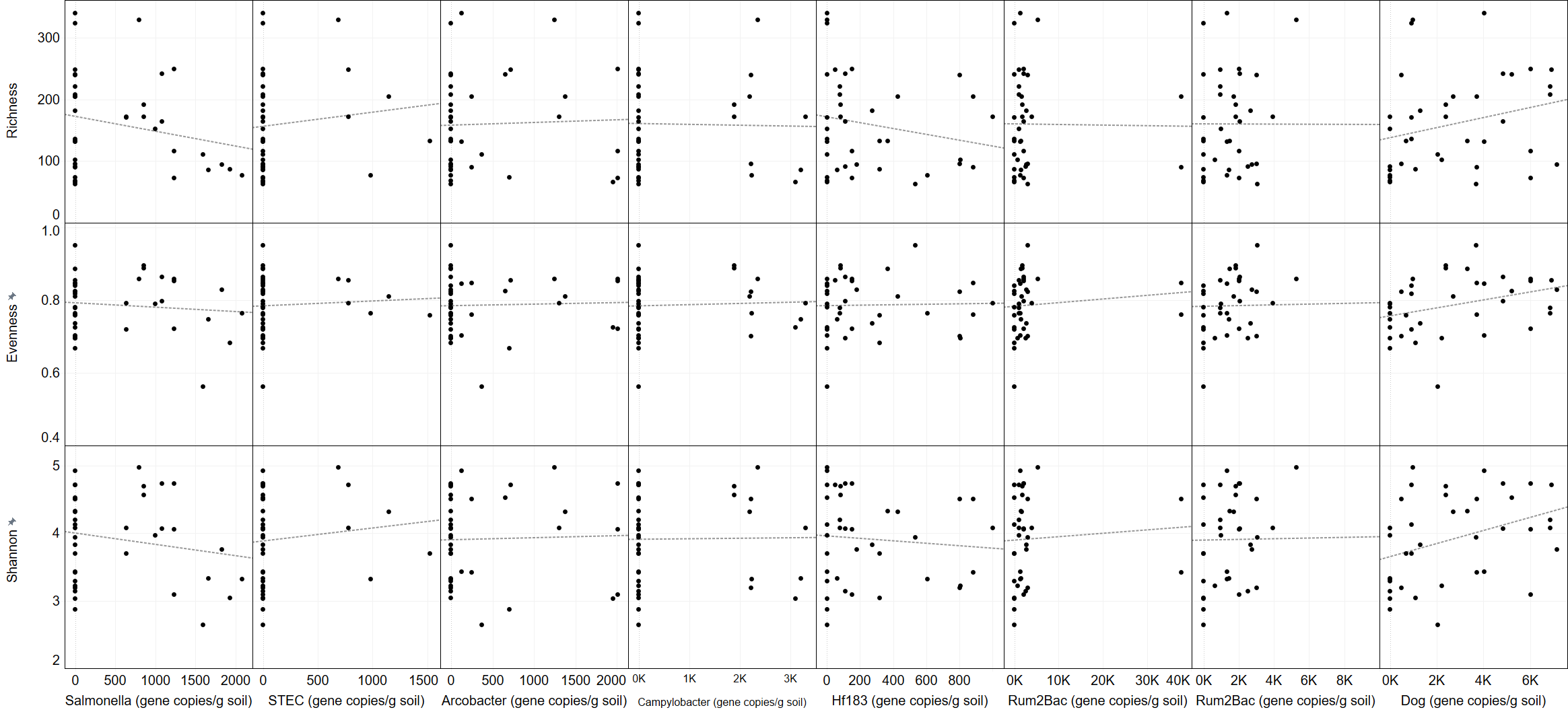


*p* > 0.05

*p* > 0.05

*p* > 0.05

*p* > 0.05

*p* > 0.05

*p* = 0.13

rho = 0.25

*p* = 0.11

rho = 0.27

*p* = 0.010

rho = 0.41

*outliers removed

*p* = 0.027

rho = 0.36

*p* = 0.024

rho = 0.38

*p* = 0.013

rho = 0.40

*

*

*

*p* > 0.05

*p* > 0.05

*p* > 0.05

*p* > 0.05

*p* > 0.05

Index

(b)

*

**
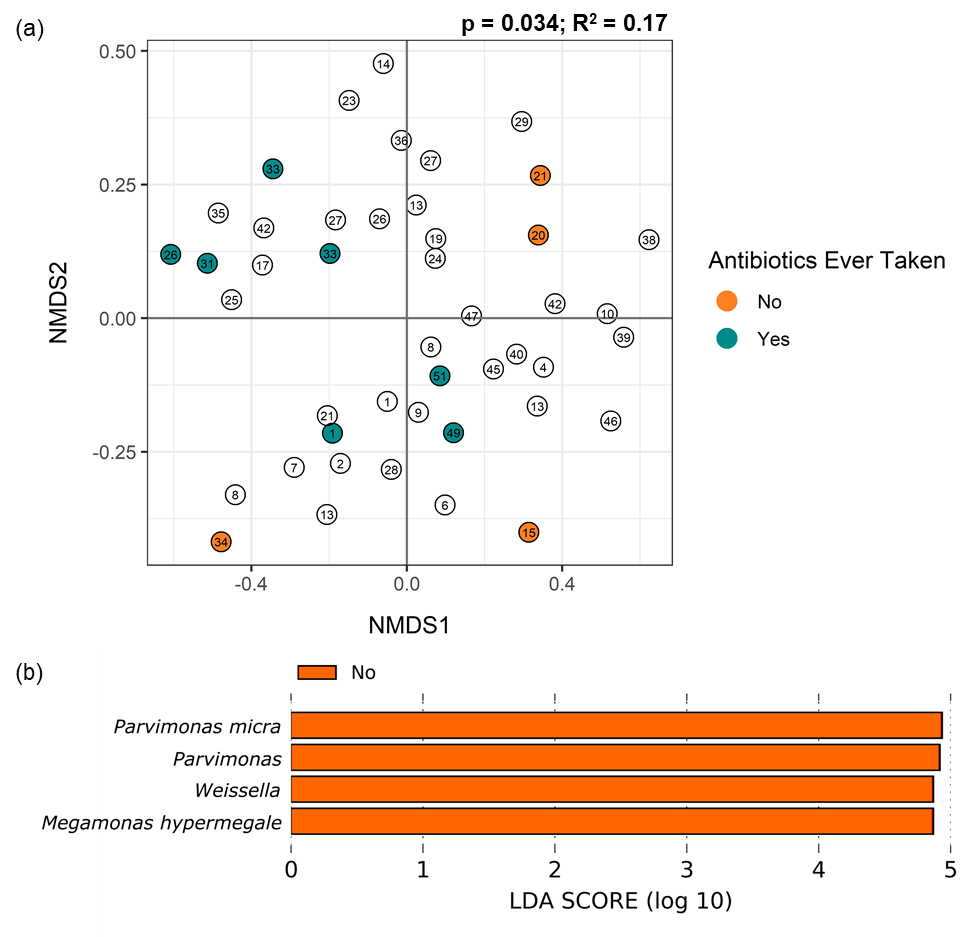
Figure S5.** a) Beta diversity of children and infants who have received antibiotics (turquoise) and have not received antibiotics (orange), plotted with Non-Metric Multi-dimensional Scaling (NMDS) ordination based on Bray Curtis dissimilarity. The number in each point indicates the household that sample was collected at. Samples that did not have a response are shown in white. (b) Differentially abundant taxa identified between children and infants who had and had not received antibiotics using linear discriminant analysis effect size (LEfSe). Bacteria and archaea that were significantly more abundant in subjects who had not taken antibiotics are shown in orange.


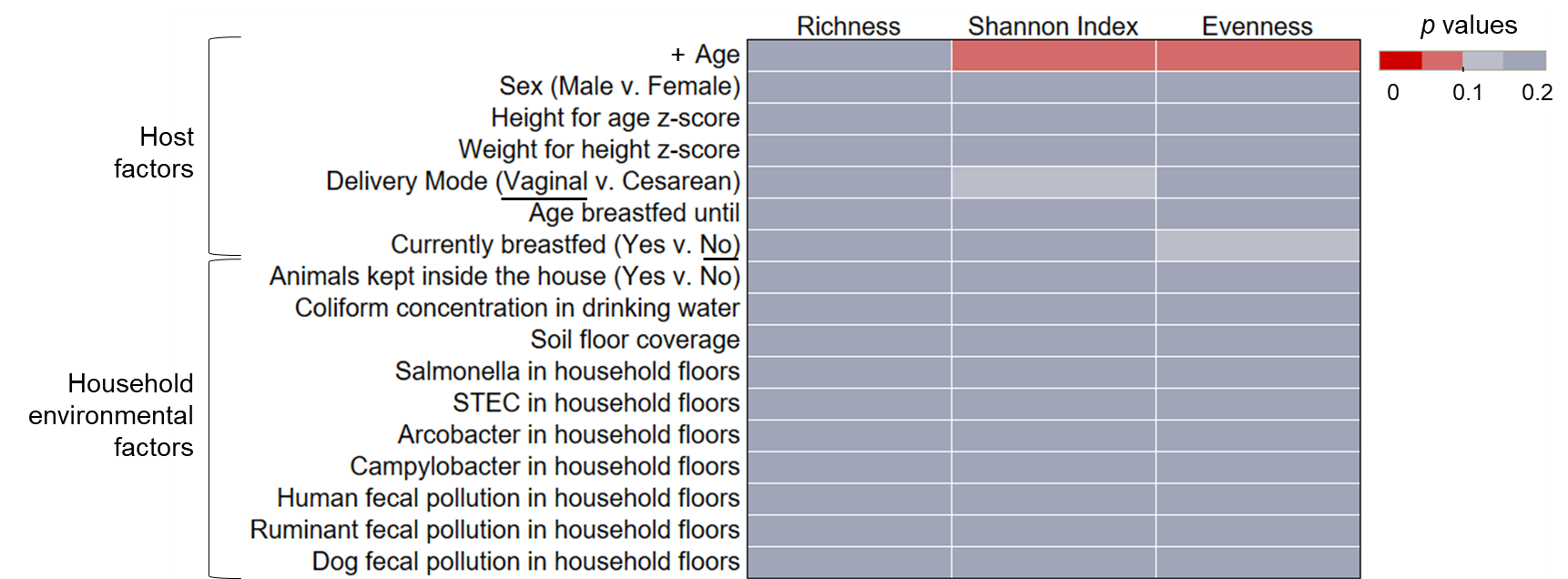


(a)

(b)


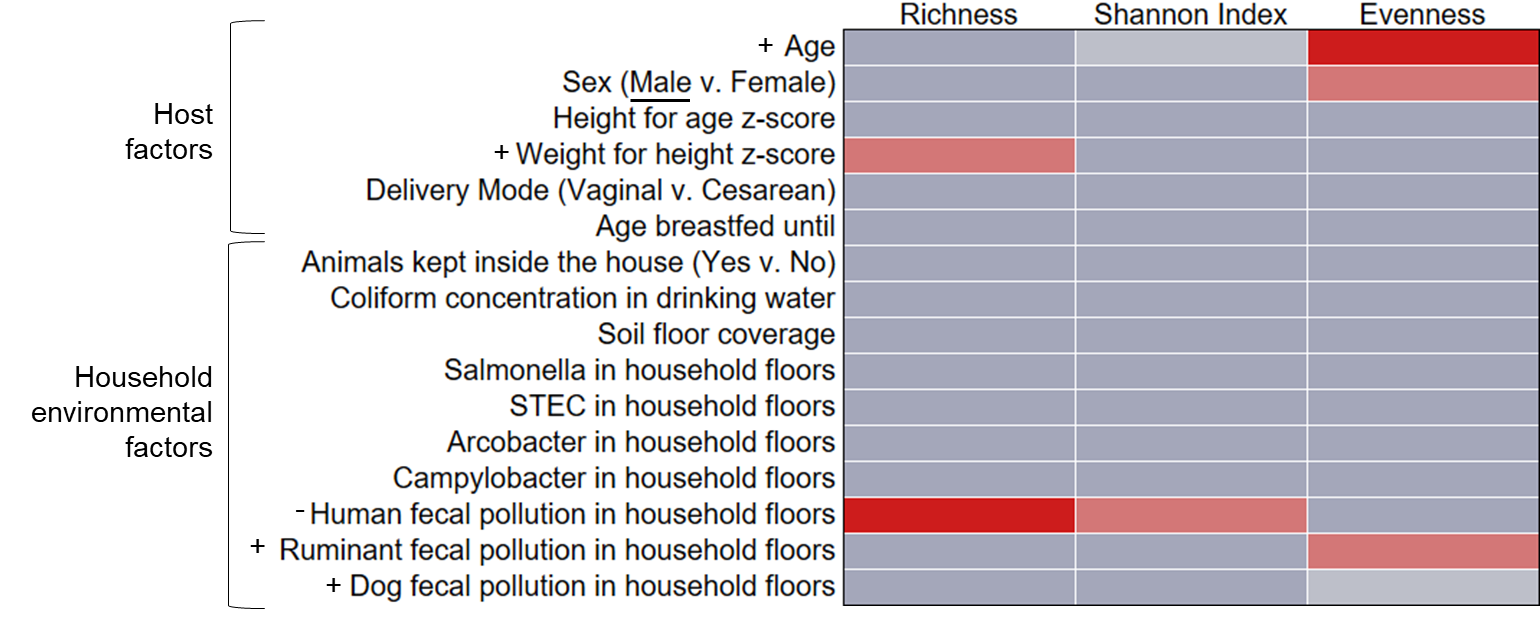

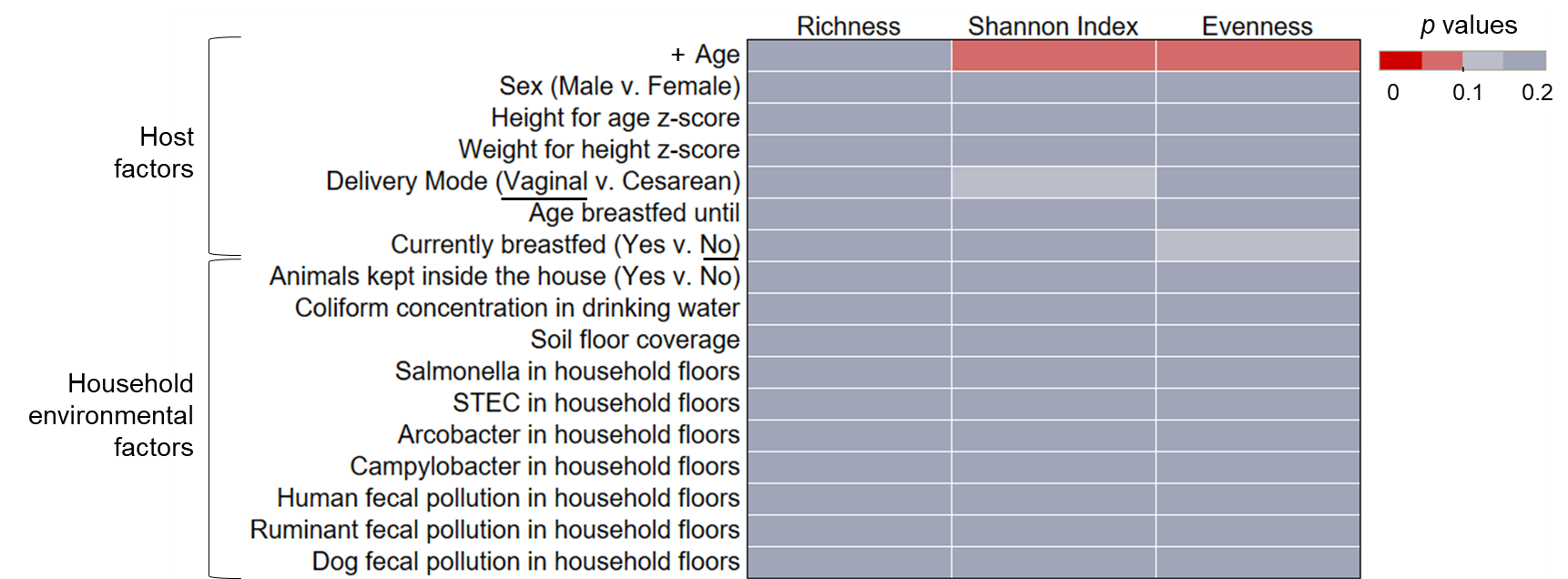

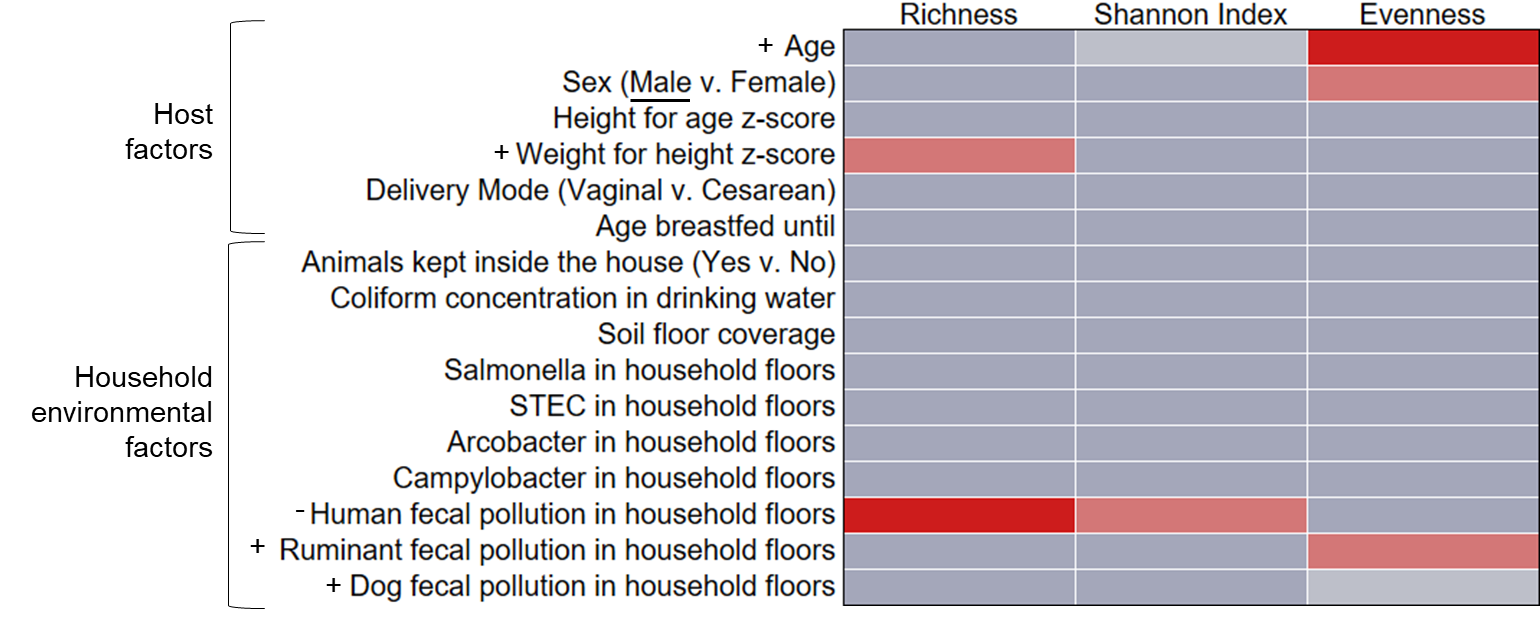


**Figure S6.** The significance of factors tested amongst alpha diversity indices of microbiome Shannon index and Pielou’s evenness in infants (a) and children (b). Significance was assessed via *p* value, with values differentiated by color. Significant values (*p*<0.05) are shown with the darkest red. For significant factors, the direction of the trend is identified in the factor label (y-axis). For correlation tests of continuous variables, the direction of the significant association is indicated with a + or -. For categorical data, the group with significantly greater diversity is underlined. All cells shaded with the darkest grey had a *p* value >0.15 (not significant, not reported).


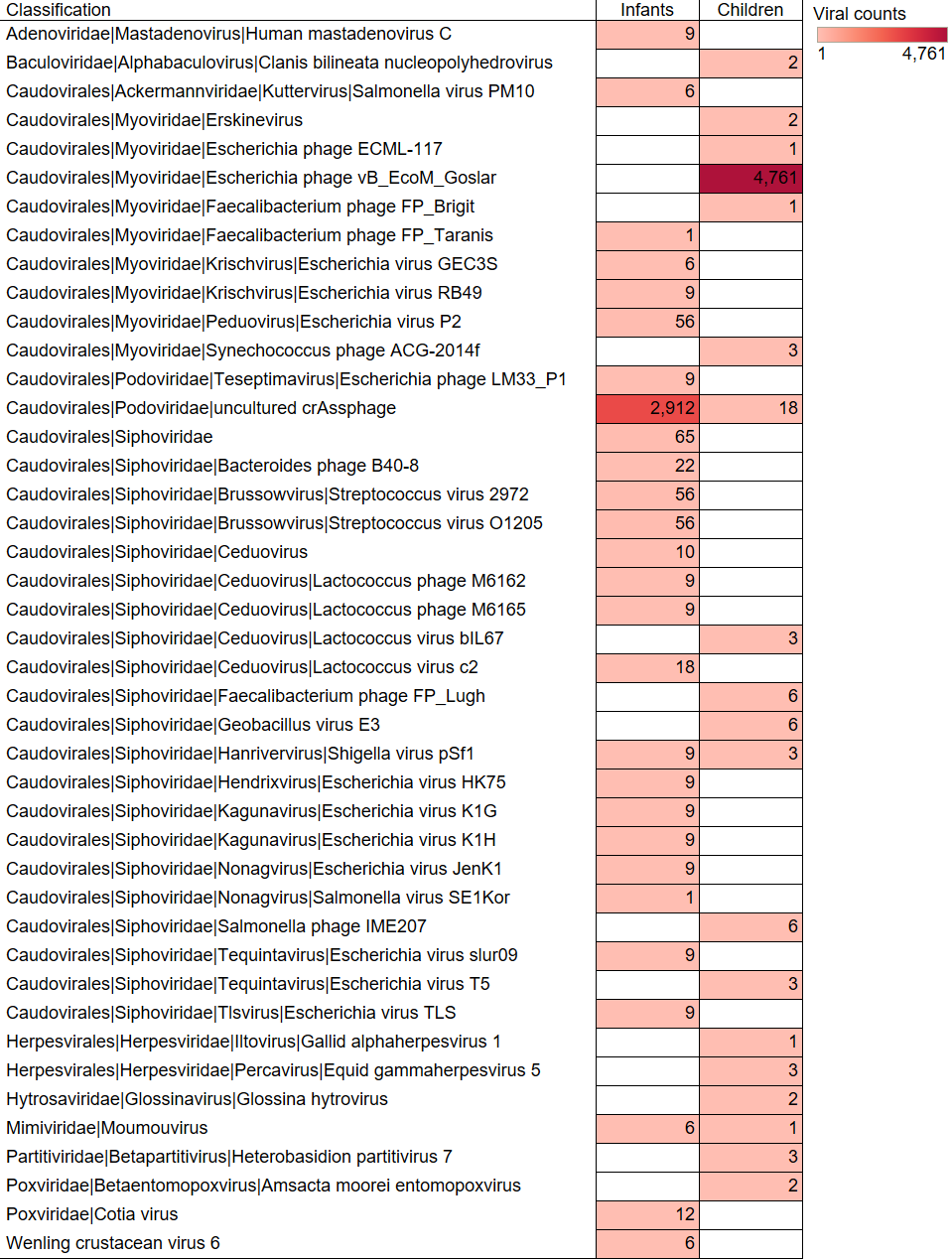
**Figure S7.** Viruses to a species level identified in infant and children gut microbiomes shown as the total number of counts.


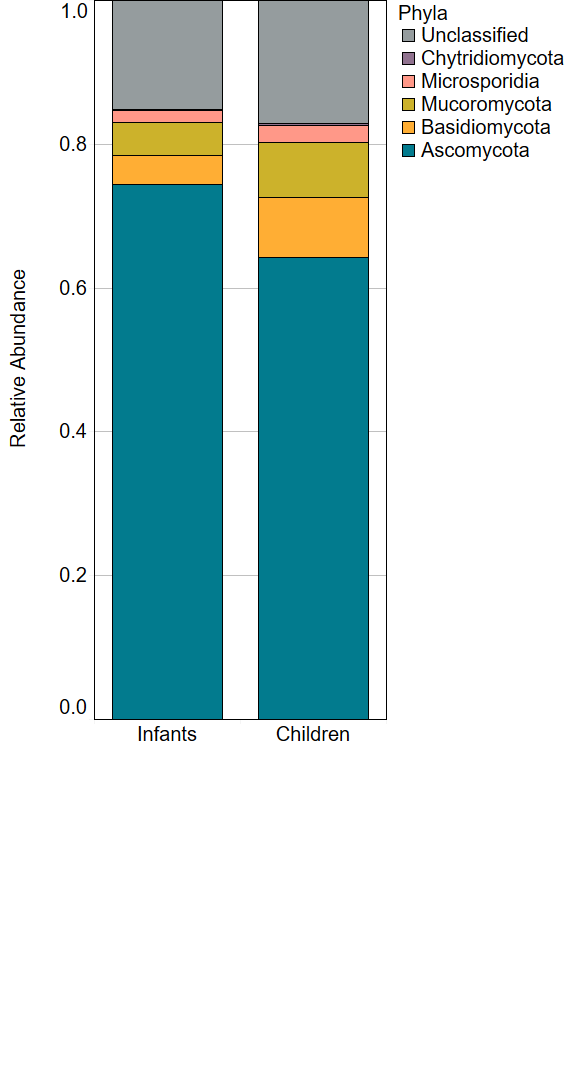


**Figure S8.** Fungal communities in infant and children gut microbiomes at a phyla level expressed as relative abundance. These were a subset of 23 samples sequenced at a greater depth than the full dataset.


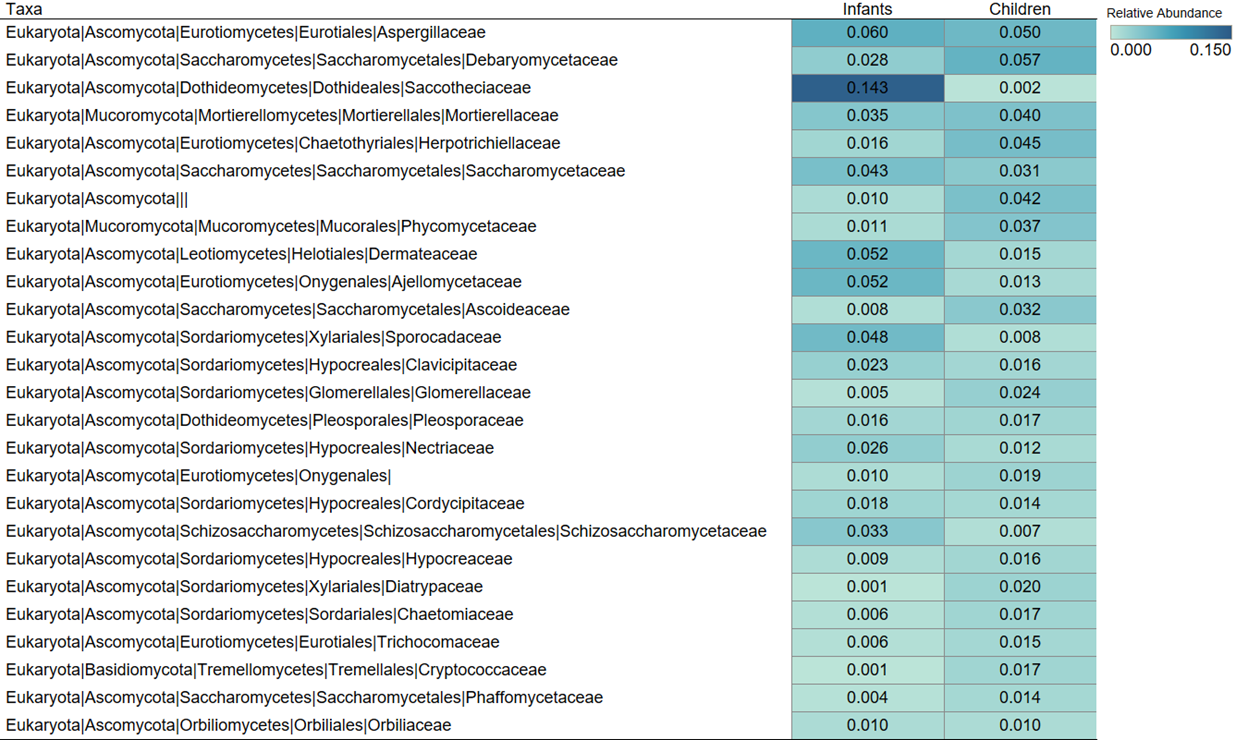


**Figure S9.** The fungal community results at a family level in the gut microbiomes of children and infants. Color and text within each cell indicate the average relative abundance of that family out of the total fungal reads.


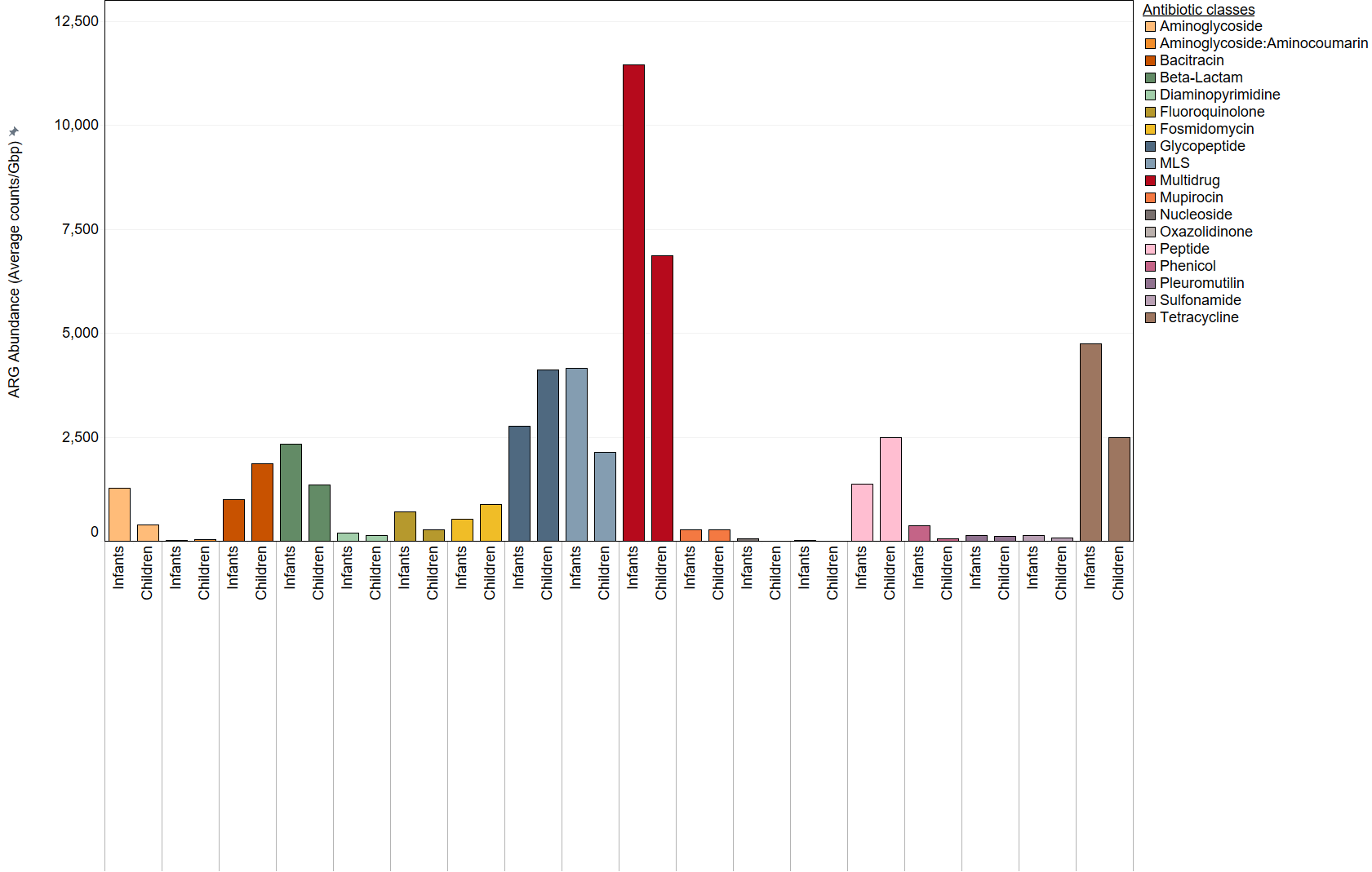


**Figure S10.** Comparison of the total normalized gene abundance (in average gene counts/gigabase pair (gbp)) of each antibiotic resistance gene (ARG) class identified in children and infants. The color key indicates the class of antibiotics to which the ARG gives resistance.


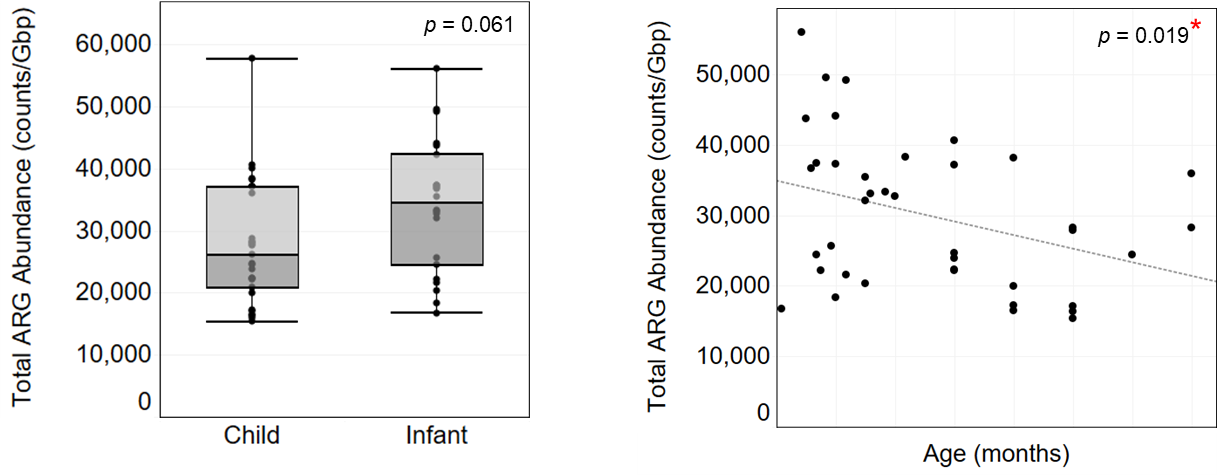


(a)

(b)


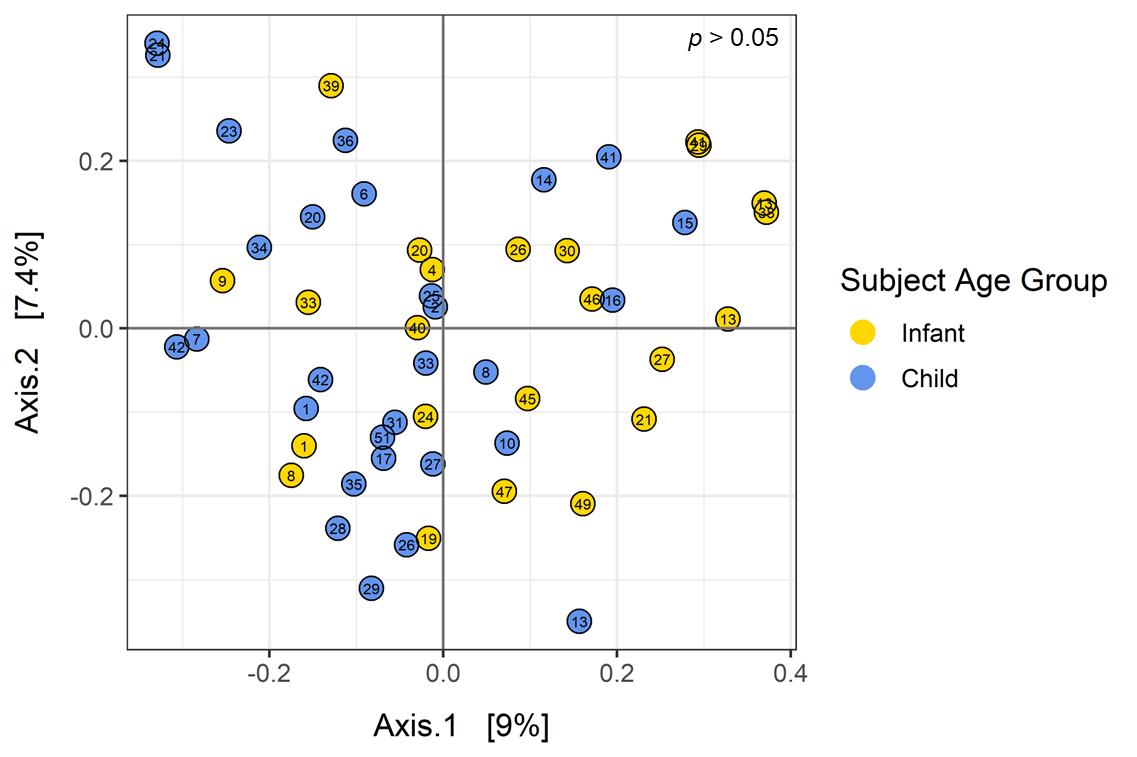


(c)

**Figure S11.** (a) The difference in the gene abundance of all antibiotic resistant genes (ARGs) (count of ARGs, normalized to sequence length (gigabase pairs (gbp)) between infant and children’s resistomes. Statistical results are shown for Wilcoxon rank sum (b) Change in ARG abundance with subject age. Spearman’s rank correlation results are shown per association. (c) Beta diversity of resistomes is plotted with Principal Coordinates Analysis (PCoA) ordination based on Bray Curtis dissimilarity. Percentages on the x- and y-axis show the variation in the data that can be explained by that axis. There were no differences in resistome structure between infants (yellow, ages 0-2) and children (blue, ages 2-6), identified with adonis tests (*p*>0.05). **p*<0.05, ***p*<0.01, ****p*<0.001

**
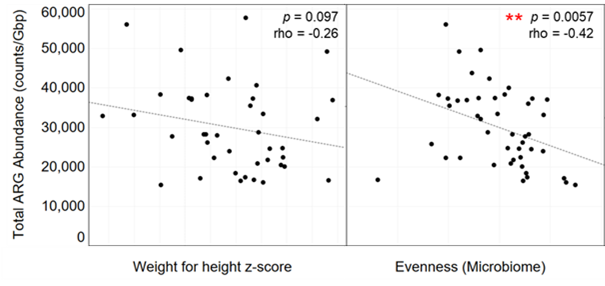
Figure S12.** Spearman correlation results for resistome abundance and numerical metadata categories in child and infant fecal samples. Correlation *p* and *rho* values are presented. ***p*<0.01


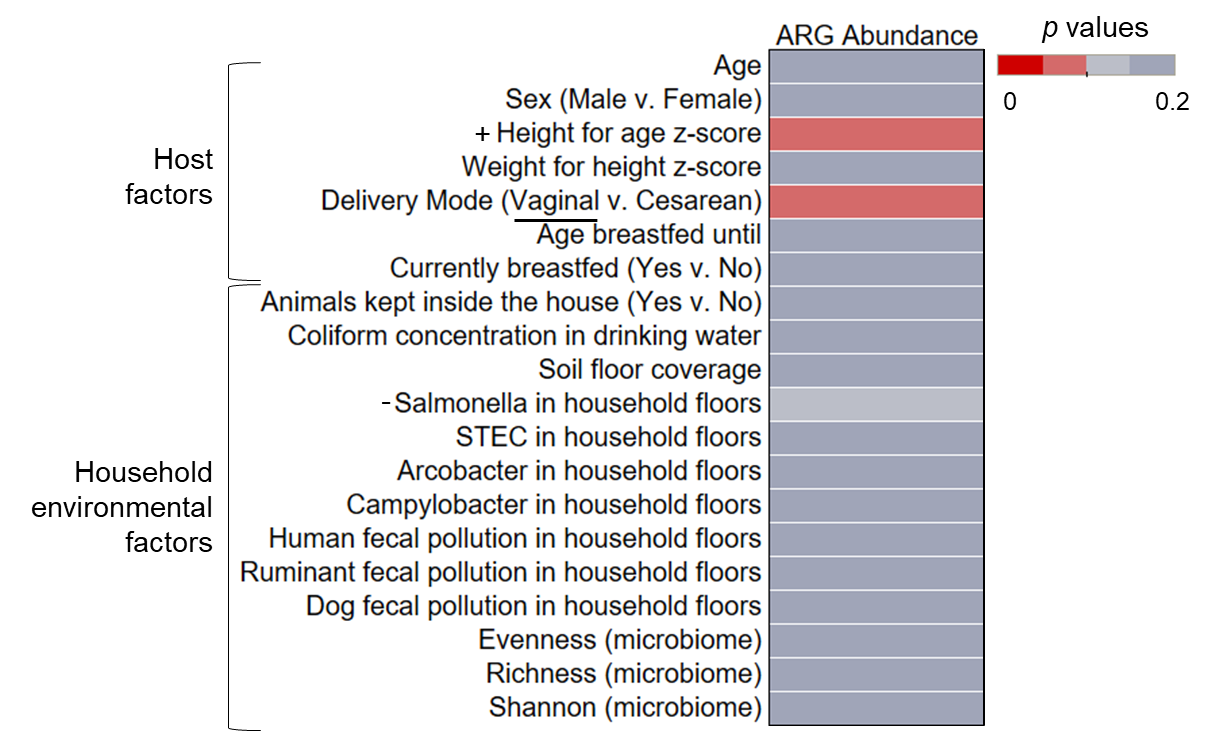


(a)

(b)


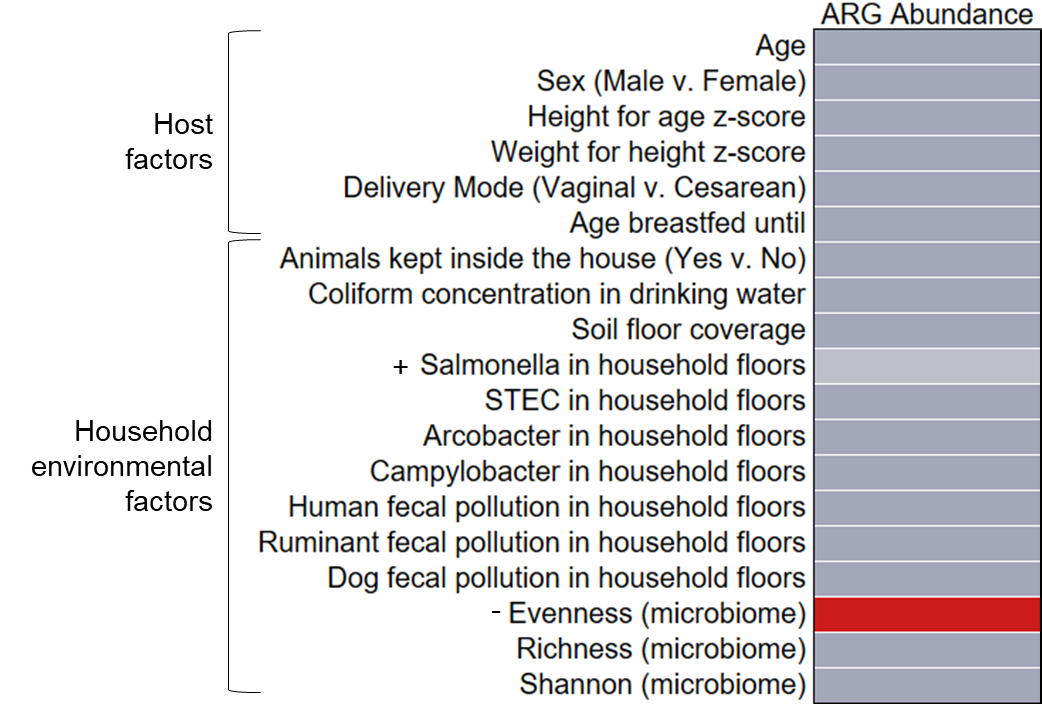


**Figure S13.** The comparison of the significance of different factors in describing total antibiotic resistance gene (ARG) abundance (normalized ARG counts/gigabase pair) in infants (a) and children (b). Significance was assessed with *p* values, which are indicated by color. Significant values (*p*<0.05) are shown with the darkest red. For significant factors, the direction of the trend is identified in the factor label (y-axis). For correlation tests of continuous variables, the direction of the significant association is indicated with a + or -. For categorical data, the group with significantly greater diversity is underlined. All cells shaded with the darkest grey had a *p* value >0.15 (not significant, not reported).


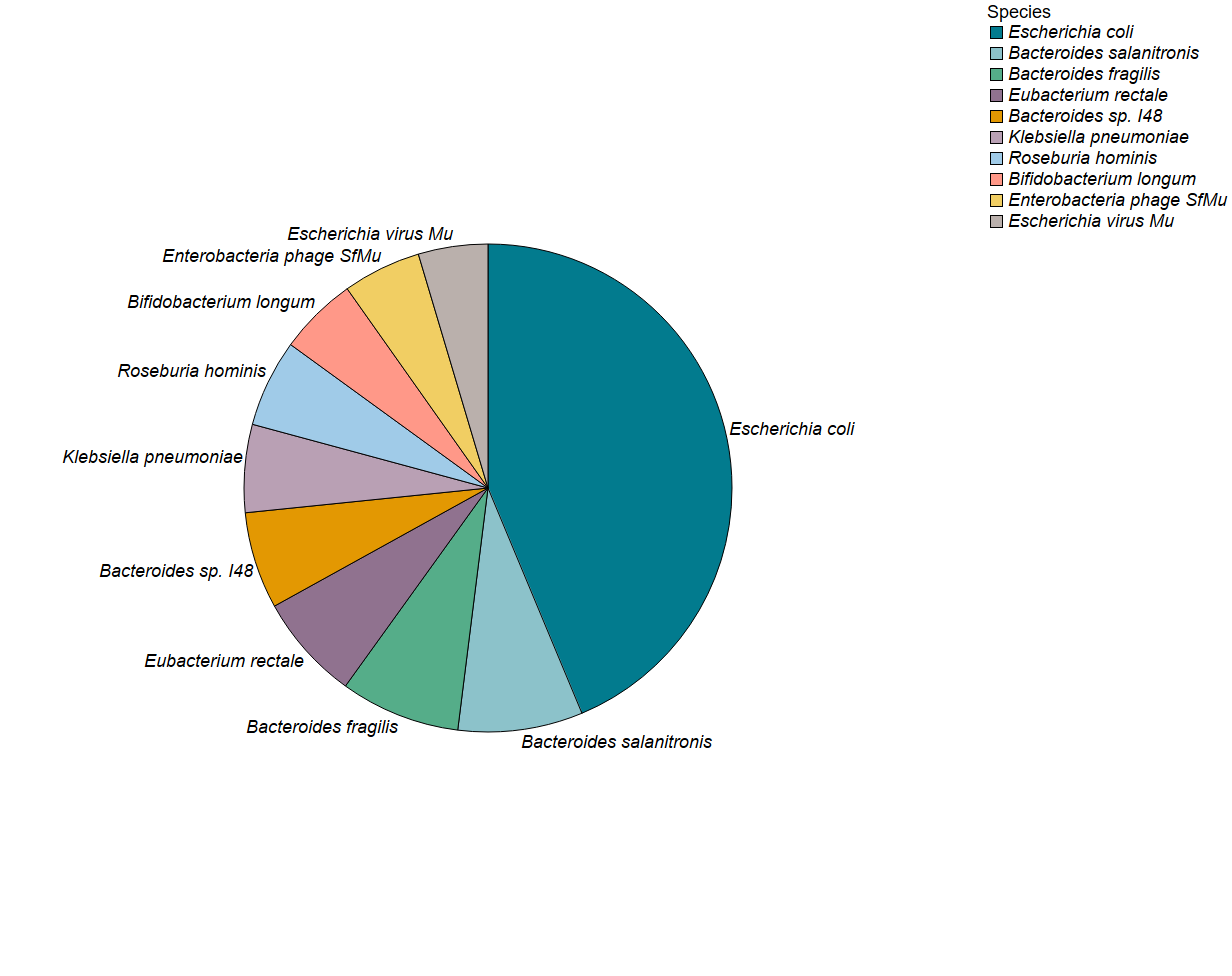

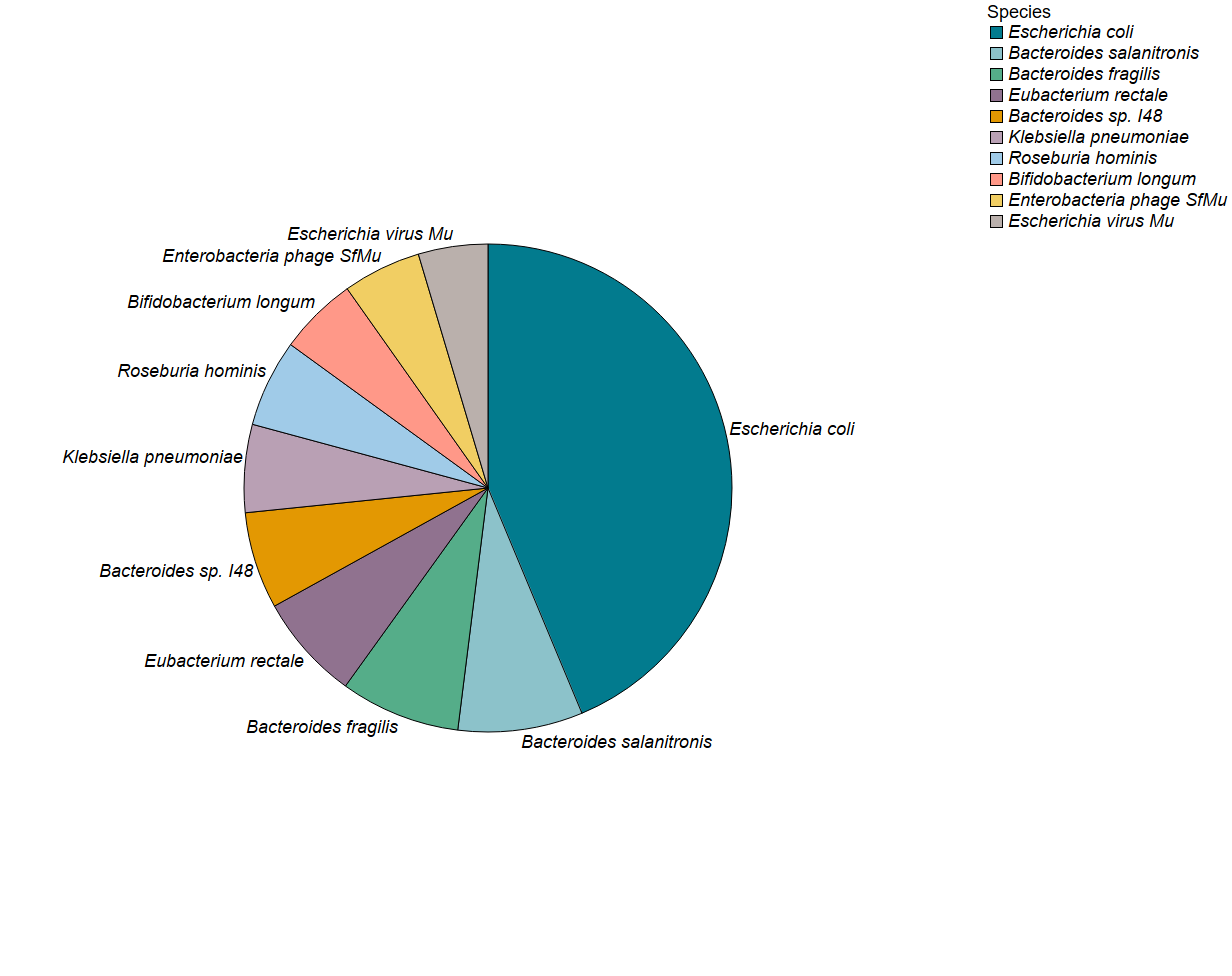


**Figure S14.** The top 10 most abundant classified antibiotic resistance gene (ARG) hosts at a species level of taxonomy in all children and infant gut samples.


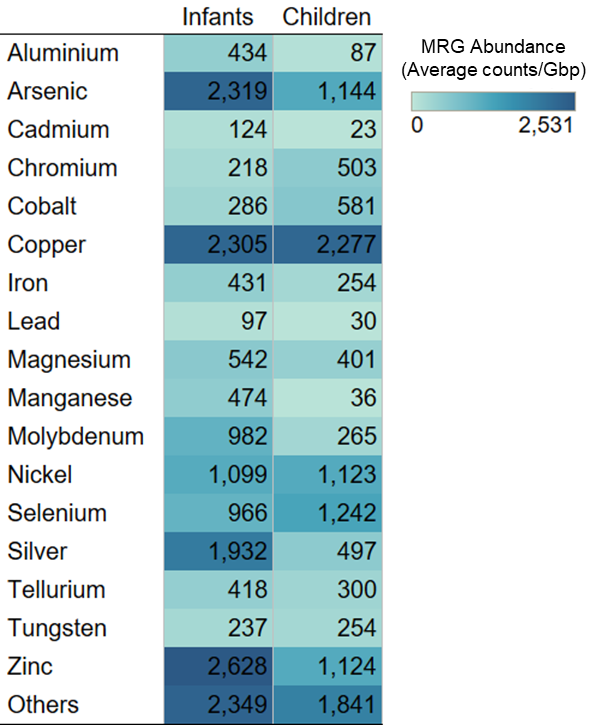


**Figure S15.** Comparison of the abundance of metal resistance genes (MRGs) in children and infants by metal. MRG abundance is indicated by the color and text within each cell and is expressed as normalized gene count/gigabase pair (gbp).


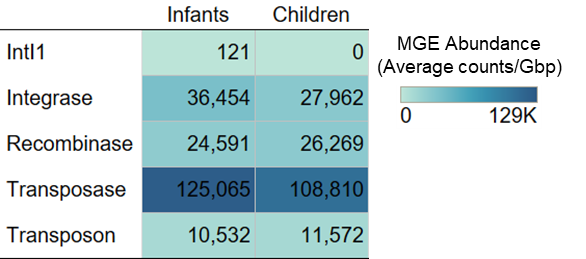


**Figure S16.** Comparison of the abundance of mobile genetic elements (MGEs) in children and infants. MGE abundance is indicated by the color and text within each cell and is expressed as normalized gene count/gigabase pair (gbp).

**References**

Callahan, B., McMurdie, P., Rosen, M., Han, A., Johnson, A., & Holmes, S. (2016). DADA2:

High-resolution sample inference from Illumina amplicon data. *Nat Methods*, *13*(7): 581-583. <https://doi.org/10.1038/nmeth.3869>

González, A., Suski, J., & Ferrús, M.A. (2010). Rapid and accurate detection of *Arcobacter*

contamination in commercial chicken products and wastewater samples by real-time polymerase chain reaction. *Foodborne Pathog Dis*, *7*(3):327-338. <https://doi.org/10.1089/fpd.2009.0368>

Green, H.C., Haugland, R.A., Varma, M., Millen, H.T., Borchardt, M.A., Field, K.G., et al.

(2014). Improved HF183 quantitative real-time PCR assay for characterization of human fecal pollution in ambient surface water samples. *Appl Environ Microbiol*, *80*(10):3086-3094. <https://doi.org/10.1128/AEM.04137-13>

Healy-Profitós, J., Lee, S., Mouhaman, A., Garabed, R., Moritz, M., Piperata, B., & Lee, J. (2016). Neighborhood diversity of potentially pathogenic bacteria in drinking water from the city of Maroua, Cameroon. *J Water Health*, *14*(3):559-570. <https://doi.org/10.2166/wh.2016.204>

Hein, I., Flekna, G., Krassnig, M., & Wagner, M. (2006). Realtime PCR for the detection of

*Salmonella* spp. In food: an alternative approach to a conventional PCR system suggested by the FOOD-PCR project. *J Microbiol Methods*, *66*(3):538-547. <https://doi.org/10.1016/j.mimet.2006.02.008>

Ibekwe, A.M., Watt, P.M., Grieve, C.M., Sharma, V.K., & Lyons, S.R. (2002). Multiplex fluorogenic real-time PCR for detection and quantification of Escherichia coli O157:H7 in dairy wastewater wetlands. *Appl Environ Microbiol*, *68*(10):4853-4862. <https://doi.org/10.1128/aem.68.10.4853-4862.2002>

Josefsen, M.H., Jacobsen, N.R., & Hoorfar, J. (2004). Enrichment followed by quantitative PCR

both for rapid detection and as a tool for quantitative risk assessment of food-borne thermotolerant *Campylobacters*. *Appl Environ Microbiol*, *70*(60):3588-3592. <https://doi.org/10.1128/AEM.70.6.3588-3592.2004>

Kildare, B.J., Leutenegger, C.M., McSwain, B.S., Bambic, D.G., Rajal, V.B., & Wuertz, S. (2007). 16S rRNA-based assays for quantitative detection of universal, human-, cow-, and dog-specific fecal Bacteroidales: a Bayesian approach. *Water res*, *41*(16):3701-3715. <https://doi.org/10.1016/j.watres.2007.06.037>

McMurdie, P.J. & Holmes, S. (2014). Waste Not, Want Not: Why Rarefying Microbiome

Data Is Inadmissible. *PLoS Computational Biology*, *10*(4):e1003531. <https://doi.org/10.1371/journal.pcbi.1003531>

Mieszkin, S., Yala, J.F., Joubrel, R., & Gourmelon, M. (2010). Phylogenetic analysis of Bacteroidales 16S rRNA gene sequences from human and animal effluents and assessment of ruminant faecal pollution by real‐time PCR. *J Applied Microbiol*,

*108*(3):974-984. <https://doi.org/10.1111/j.1365-2672.2009.04499.x>

Quast, C., Pruesse, E., Yilmaz, P., Gerken, J., Schweer, T., Yarza, P., . . . Glöckner, F.

(2013). The SILVA ribosomal RNA gene database project: improved data processing and web-based tools. *Nucleic Acids Res*, 41, D590-D596. <https://doi.org/10.1093/nar/gks1219>

Weiss, S., Xu, Z.Z., Peddada, S., Amir, A., Bittinger, K., Gonzalez, A., … & Knight, R.

(2017). Normalization and microbial differential abundance strategies depend upon data characteristics. *Microbiome*, *5*:27. <https://doi.org/10.1186/s40168-017-0237-y>
